# Probabilistic Prediction of Contacts in Protein-Ligand Complexes

**DOI:** 10.1371/journal.pone.0049216

**Published:** 2012-11-14

**Authors:** Riku Hakulinen, Santeri Puranen, Jukka V. Lehtonen, Mark S. Johnson, Jukka Corander

**Affiliations:** 1 Department of Natural Sciences, Mathematics and Statistics, Åbo Akademi University, Turku, Finland; 2 Department of Biosciences, Åbo Akademi University, Turku, Finland; 3 Department of Mathematics and Statistics, University of Helsinki, Helsinki, Finland; Semmelweis University, Hungary

## Abstract

We introduce a statistical method for evaluating atomic level 3D interaction patterns of protein-ligand contacts. Such patterns can be used for fast separation of likely ligand and ligand binding site combinations out of all those that are geometrically possible. The practical purpose of this probabilistic method is for molecular docking and scoring, as an essential part of a scoring function. Probabilities of interaction patterns are calculated conditional on structural x-ray data and predefined chemical classification of molecular fragment types. Spatial coordinates of atoms are modeled using a Bayesian statistical framework with parametric 3D probability densities. The parameters are given distributions *a priori*, which provides the possibility to update the densities of model parameters with new structural data and use the parameter estimates to create a contact hierarchy. The contact preferences can be defined for any spatial area around a specified type of fragment. We compared calculated contact point hierarchies with the number of contact atoms found near the contact point in a reference set of x-ray data, and found that these were in general in a close agreement. Additionally, using substrate binding site in cathechol-O-methyltransferase and 27 small potential binder molecules, it was demonstrated that these probabilities together with auxiliary parameters separate well ligands from decoys (true positive rate 0.75, false positive rate 0). A particularly useful feature of the proposed Bayesian framework is that it also characterizes predictive uncertainty in terms of probabilities, which have an intuitive interpretation from the applied perspective.

## Introduction

Atomic level structures are an important source of information for inferring functional aspects about macromolecules and ligands binding to them. For instance, this is illustrated by the substantial amount of existing algorithms and structural data modeling software created for molecular docking and scoring purposes [1], [2], [3], [5], [6]. The Protein Data Bank (PDB) [7] offers the central public access to macromolecular structure files.

Although there is already a large amount of structural data available, it is by no means straightforward to model it reliably. There are several reasons for this, such as the inevitable errors present in experimental results and the “averaging” nature of the measurement process used in the construction of x-ray diffraction data. Moreover, along the conversion from a measurement to a structural coordinate file, several computational approximations and the subjective choices of experimentalists will influence the final outcome. Among the latter sources of variability, two major issues are flexibility of the molecules and computational constraints implemented in the refinement process. The first one is related to thermal motion and static disorder, and the second to biochemical *a priori* information that is always used in the refinement of a structure to create a coordinate file [6], [8]. These are accompanied by crystal packing effects, which also originate from the flexibility of the molecules, uncertainty in orientation and location of small molecules, including water.

It can be argued that for addressing the above-mentioned issues, statistical modeling provides the most promising approach, given its ability to capture uncertainties and errors in data. To meet these goals we introduce a Bayesian statistical method for evaluating atomic level 3D interaction patterns of protein-ligand contacts. Our work is motivated by the previous findings in Rantanen et al., [1], [2], [3] which showcased the usefulness of this kind of a multidisciplinary approach. However, given computational speed related constraints, it has not been possible to pursue these previous Bayesian methods further in contact preference exploration. Therefore, the method discussed here focuses on providing rapid means of computing, together with adjustability and robustness of the statistical model. The latter aspect refers in this context firstly to the constraint that two points in close proximity to each other (with respect to the system size) should not get very different contact preference hierarchies without an easily tractable reason. Secondly, in terms of robustness, the preference prediction model has to be balanced between adhering too closely to the possibly biased overall number of different types of contact atoms in the training data set and using a sole comparison of the probability densities defined for each contact atom type of a molecular fragment. Finally, the adjustability is concerned with both the model structure and the chemistry-based classification of molecular fragments. In our illustrations we consider 24 molecular fragment and 13 contact atom types, exhibiting interactions like hydrogen bonding, dispersion (e.g. aromatic-aromatic) and interactions between charged groups.

The main purpose of this paper is to show how a Bayesian statistical modeling approach can be utilized to make naturally ranked predictions about contact preferences, such that the model itself can be flexibly updated in the presence of novel data and other auxiliary information. Basically, this method is developed to retrieve information to be used in a knowledge-based scoring function. There exist several well performing scoring functions [9], [10] that utilize the experimental knowledge through inverse Boltzmann relation from statistical thermodynamics [11]. These functions depend only on distance between atoms, e.g., a ligand atom and a binding site atom. Our method differs from them in that also directional information is incorporated in the model, which has been shown in case of hydrogen bonding to still significantly improve evaluation of binding energetics from experimental data [12].

Three basic scoring function tasks have been defined [9], of which enrichment of ligands was tested with our method. The test was done through separating catechol-O-methyltransferase (COMT) ligands from decoys using logistic regression on a set of 27 small molecules having similar properties. The receiver operating characteristics (ROC) [13] for the results show that the probabilistic contact preferences give reliable information about the relative affinities in intermolecular contacts. These probabilities can be applied to intramolecular contacts as well. In practice, they are used as part of a molecular docking and scoring routine. The method described in this paper will be integrated as a functionality in the molecular modeling environment BODIL [4].

The structure of the article is as follows. First, data collection and the modeling approach are described. Thereafter, results from several case-studies are presented. Last, implications of the results and some future prospects are discussed.

## Materials and Methods

### 0.1 Data collection and processing

We used PDB as the main source of data in this work. Training data for the model was collected from a set of approximately 28000 structure files published before January 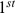 2009. The files were selected using the criteria presented in Table 1. A reference dataset for model validation was selected under the same criteria as the training set and contained 10361 structure files published between February 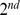 of 2009 and 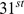 August 2011. X-ray structures form the biggest group of data present in PDB. The bulk of a structure file is the coordinate section, but there is also chemical and biological information, interpreted as metadata, which is necessary for constructing a sensible predictive model. The type of metadata that is most directly deducible from the experimental observations is the atom type. The atom type classification can be considered sufficiently reliable for the higher resolution (<2.5 Å ) structures, however, with at least one exception, which corresponds to the nitrogen (N) and oxygen (O) atoms in a carbamoyl group (-CO-NH_2_). In this group, O and N cannot be distinguished solely on the basis of x-ray diffraction data, because of the symmetric structure of the group and very similar electronic densities around both O and N. This is a prime example of a regularly encountered error in the metadata, which, however, can be corrected by reversing the coordinates of O and N. The ligand metadata would impose this error when for instance hydrogen bonding with the ligand would require an acceptor (O) contact, but a donor (-NH_2_) contact is given a closer coordinate location in the structure file.

**Table 1:** Criteria for selecting structures from PDB.

Has ligands:	Yes
Contains: Protein,DNA,RNA	Yes, No, No
Experimental method:	X-ray diffraction
Min. resolution	2.5 Å

A considerably more difficult problem to handle is the influence of the constraints used in the refinement of the protein structure from experimental data. These constraints generate some unreliability in the coordinates, because only conformations with restricted geometries are allowed for the amino acid chain, which together with limited resolution can lead to artificially distorted conformations of ligand structures. In practice this means that the refinement involves fitting an alleged structure to the experimentally determined electron density map, which does not define all structural features uniquely, especially when the resolution is low [8].

Molecular fragments of pre-defined types (see Tables 2 and 3) were searched from coordinate ligand structure files in PDB. The search was based on atom types, chemical connectivity and geometry, and the identified fragments were then labelled for use in the extraction of coordinate data from protein structure files. To obtain unique fragment orientations, atoms from within a functional group were, when possible, chosen for the fragment definitions. In order to build a predictive model, the set of 24 fragment classes in Table 2 was used while collecting a dataset of approximately 70,000 contacts, representing the 13 contact atom types, i.e. target classes in Table 3.

**Table 2:** Fragment classes used in this study.

Class	Description
f2	Hydroxyl oxygen bonded to a non-planar aliphatic structure
f3	Hydroxyl oxygen bonded to an aromatic structure
f5	Carbonyl oxygen (excluding those belonging to f9 and f10)
f6	Oxygen of a carboxyl group
f7	Carbamoyl oxygen
f8	Oxygen bonded to a phosphate group
f9	Amide group oxygen bonded to a non-aromatic structure
f10	Amide group oxygen bonded to an aromatic structure
f11	Secondary carbon in an aromatic structure
f12	Secondary carbon in a non-aromatic structure
f13	Primary carbon (with one hydrogen)
f17	Fluorine bonded to an aromatic structure
f18	Fluorine bonded to a non-aromatic structure
f20	Chlorine bonded to an aromatic structure
f21	Chlorine bonded to a non-aromatic structure
f22	Nitrogen in an aromatic structure (without a substituent)
f23	Nitrogen in a non-aromatic planar ring structure (without a substituent)
f26	Amino (primary) nitrogen singly bonded to a non-aromatic structure
f27	Amino (primary) nitrogen bonded to an aromatic structure
f29	Amino (primary) nitrogen singly bonded to a planar structure
f34	Bromine bonded to an aromatic structure
f35	Bromine bonded to an aliphatic structure
f36	Iodine bonded to an aromatic structure
f37	Iodine bonded to an aliphatic structure

Main forms of intermolecular interaction for these fragment types are hydrogen bonding, dispersion, charged group based electrostatic and halogen bonding. The fragment classification was partly adopted from the previous work of Rantanen et al. (see Introduction) while some classes were excluded. To maintain consistency of the notation for easy comparison with earlier work, the classes are not renumbered.

**Table 3:** Classification of contact atoms, or targets.

Class	Description
C3	Carbon of a methyl group
C4	Alpha carbon
C5	Carbon in an aromatic structure
C6	Sulfur of a thioether group
C7	Sulfur of a thiol group
C8	Nitrogen of an amide group
C9	Nitrogen of indole, imidazole and guanido groups
C10	Nitrogen of an amino group
C11	Oxygen of a carboxamide group
C12	Oxygen of a carboxyl group
C13	Oxygen of a hydroxyl group
C14	Main chain carbonyl oxygen
C15	Main chain amide nitrogen

The target classification was adopted from the previous work of Rantanen et al. (see Introduction) while some classes were excluded. As in Table 2, the classes are not renumbered for the consistency of notation.

Regarding the contact classes in Table 3, for example, the class **C3** represents a pure van der Waals contact [14] and class **C4**, a hydrogen donor in a possible weak hydrogen bond in addition to a van der Waals contact [15], [16]. Aromatic carbons (**C5, f11**) can participate in both of the typical **C3** and **C4** interactions [17]. Halogen bonds have a role in biological processes [18] and therefore Fluorine [19], Chlorine, Bromine and Iodine are considered as so called fragment Main-atoms, as shown in Table 2. The target atoms in proteins, identified with three distance criteria (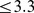 Å for alleged H-bonds and charged groups, 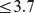 Å for probable dispersion and 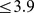 Å for halogen bonds), were classified during the search using three criteria: 1) element, 2) amino acid residue and 3) side or main chain atom. The interaction was defined as between a fragment type and target type, or between nuclei, mediated by protons and/or electrons.

A fragment was defined by an atom triple: Main-atom, Atom1 and Atom2, and at least the Main-atom was given the following characteristics: element, covalent bond count, aromaticity and possibly functional group, see Tables 2 and 3. These characteristics were used in collecting data from PDB, resulting in coordinates with metadata. The aromaticity of an atom was decided using PDB Ligand Dictionary through PDBeChem [20].

In addition to classification, target atoms have to be put in one coordinate system, i.e. fragments are superimposed. This was done using an elementary translation-rotation: first the database coordinates of the Main-atoms were translated to origin, which creates a new dataset (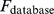 below in eq. 1), and then a rotation operation was defined to connect the fragments reference frame to a common coordinate system. This requires solving the following matrix equation:
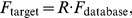
(1)where 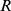 refers to a 3
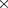
3 rotation;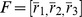, 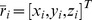 and 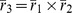, i.e., 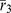 is the cross product of 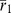 with 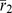.

Thus, we have the equation,
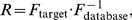
(2)which was solved for each fragment. The resulting 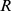 was then used in the translation-rotations of the respective target atom position vectors to the common coordinate system. When the data is collected as mentioned above, as well as classified and coordinate systemized in this manner, the process results in a collection of three dimensional distributions of points that present measured relative positions of specified atoms with respect to specified fragment types. These distributions were then modeled with 3D probability densities described below.

### 0.2 Statistical modeling

To obtain predictive distributions for contact preferences we utilize a Bayesian framework where the observed 3D coordinates in the training data are modeled with interconnected parametric 1D densities, such that the parameters are provided *a priori* uncertainty characterizations in terms of probability distributions. The prior distributions enable regulation of parameter estimates in order to prevent them from depending solely on the observed data, which is desirable especially under the circumstances where the data generation process is known to harbor intrinsic biases. Also, regularization of model parameter estimates with the prior information is most crucial when certain class pairs have only very sparse training data, in which case unsmoothed estimates can be strongly biased.

**Figure 1:**
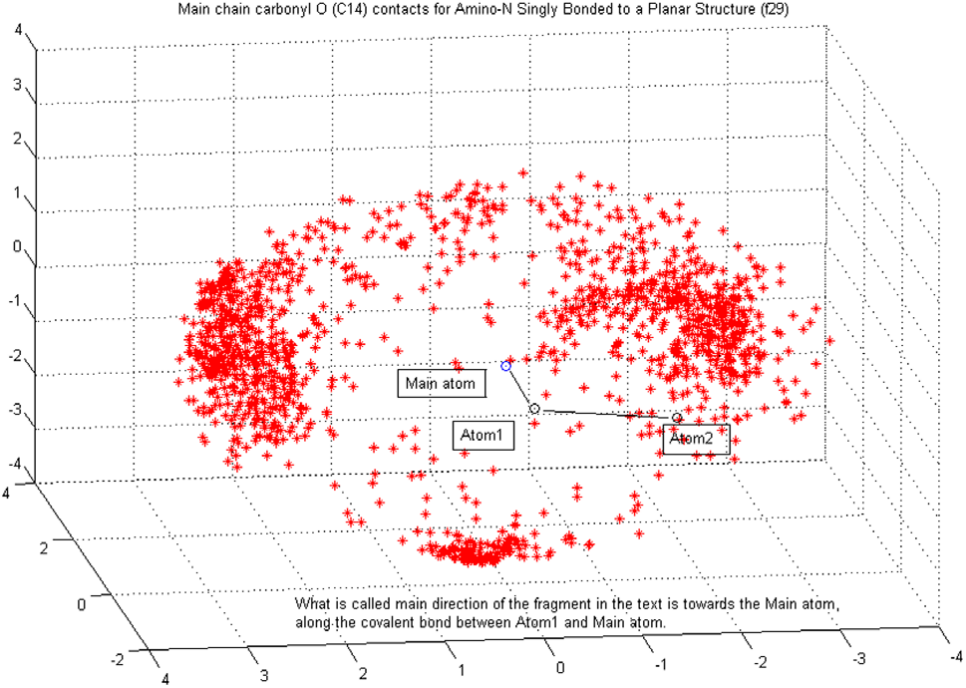
Contact atom or target cloud. It is formed by main chain carbonyl oxygens (**C14**) around fragment type (**f29**) (amino nitrogen singly bonded to a planar structure). In the reference frame where targets are modeled, polar angle value 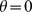 corresponds to what is in this paper called the main direction of the fragment — the direction of the vector from Atom1 to Main-atom, and 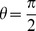 corresponds to the plane that includes Main-atom in the origo and to which the main direction is perpendicular. Azimuthal angle 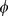 measures angular deviation from the plane of the fragment, so that the center of the smaller cluster below the fragment is (in the model frame) approximately in direction 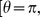 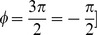. A fragment is defined by determining the characteristics of an atom triplet: Main-atom, Atom1 and Atom2. Main-atom is covalently bonded to Atom1, and Atom1 is covalently bonded to Atom2. Chemical properties of the Main-atom primarily determine the class of a fragment.

**Figure 2:**
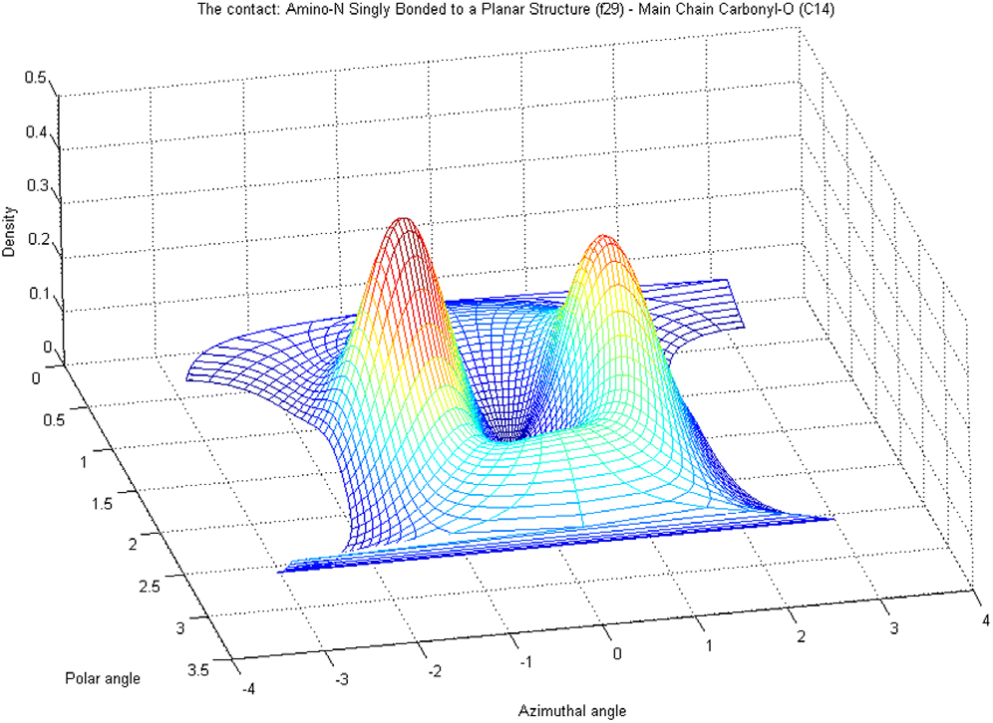
Probability density modeling the target cloud of **Figure 1**. It is depicted in the same reference frame with the density. They can be interpreted as overlayed such that elevations in the density correspond to dense areas in the cloud of data points. The main direction of the fragment, as described in the caption of Figure 1, is defined by 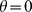 in the reference frame of the model, but corresponds in this figure to 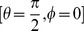, where 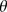 is the polar angle and 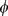 is the azimuthal angle.

The core distribution we utilize for characterizing coordinate variability is the von Mises-Fisher distribution (vMF) which is widely applied for modeling directional data. Separate probability densities for all three coordinates were necessary in order to capture the properties of the target atom clouds in a uniform setting (details provided below). Spatially the most complex (multimodal) observed differences in target atom distributions are found around the main direction of the fragment, and to a somewhat lesser extent with respect to distributions of polar angle, i.e. angular deviation from the main direction, see Figures 1 and 2. The distance distributions are given *a priori* as many modes as the corresponding polar angle distributions have, though in most cases they practically form a unimodal density, but not always. This is explained more thoroughly later in this section. The variables and parameters of the densities used in our work are specified in Tables 4 and 5.

**Table 4:** The spherical polar coordinates.

Symbol	Variable
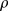	distance
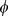	azimuthal angle
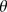	polar angle

**Table 5:** Parameters for the model densities.

Symbol	Description	Treated as
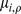	Mean of Normal density for the	Random variable
	distance ( 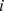 as below)	
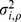	Variance of Normal density for the	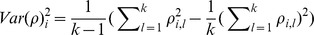 ,
	distance ( 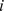 as below)	for 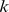 observed distances.
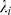	Expected direction, 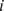 :th von Mises	Random variable
	mixture component for 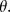	
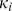	Concentration parameter, 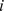 :th vonMises	Random variable
	component for 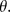	
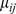	Mean, j:th Normal mixture component	Random variable
	for 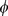 , related to i:th von Mises mixture	
	component.	
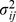	Variance, 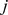 :th Normal mixture component	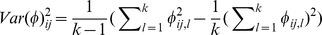 ,
	related to 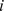 :th von Mises mixture component.	for 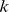 observed angles.

Let 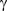 denote a generic set of parameters specifying a density in a 3D space. Then, the probability density in spherical polar coordinates 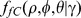 for fragment class 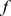 and target class 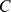 is assumed to be of the piece-wise defined form
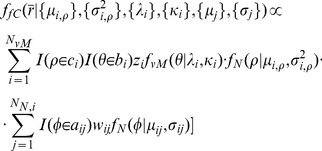
(3)where 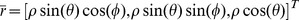, the 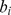 divide 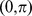 and 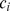 divide 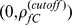 to non-overlapping intervals. The limit 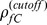 is the maximum distance used in collecting the target atom locations for a fragment class and target class pair (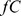). The intervals 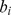 and 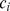 are associated with weight 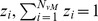. The 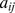 then, are non-overlapping intervals of 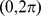 associated with the weights 
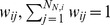
 (see below) and the different density components and parameters are defined using conventional nomenclature as:
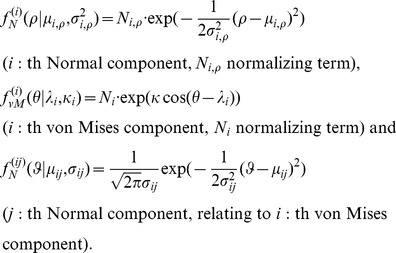
(4)
Equations 4 show the forms of the densities in the particular coordinate system, or reference frame, that is used for modeling and in which the main direction of the fragment coincides with the positive z-axis. A likelihood function is obtained as a product from the values of the components of the density (eq. 3) for 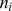 (or 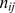) points representing each of the included regions in the sample space,
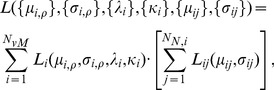
(5)where
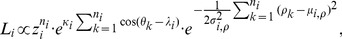
(6)

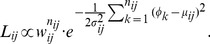
(7)


The structure of the density (eq. 3) was chosen based on investigations of the forms of the target atom clouds. For example, use of normal densities for the distance data was supported by large *p*-values in Kolmogorov-Smirnov normality test and the numbers of components needed in the angle dependent part of the density (i.e. 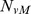 and 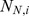) were automatically chosen based on frequency distribution of binned data. This was done for both angles (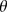 and 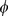) by connecting the heights of the adjacent bars of the histogram, creating a sequence of values, in which every change of sign corresponds to a local minimum or a maximum. The number of maxima was restricted to the interval [1], [5], was used to represent the number of modes in the density. The number of maxima was restricted by either reducing or increasing the number of bins in case the result would be outside the given interval.

In the separation of variables, azimuthal angle and distance are conditioned on a polar angle interval, see the equations in (4). The angular part reflects arc-like structures around the main direction of the fragment (for examples of this see last paragraph of section Examples 3 and 4). The angular deviation from the main direction is the leading variable in the sense that a multimodal azimuthal angle distribution (i.e. around the main direction) and a unimodal distance distribution are defined separately inside each polar angle segment. The idea behind this is that the peak of a polar angle density is an indicator of the strength of the interaction between a fragment and a target. The smaller the angle, the stronger the interaction, and if there is any effective multimodality in the distance density, the modes should coincide with the modes of the polar angle density. The azimuthal angle distribution thus completes the directional structure within each polar angle mode.

The uncertainties of the distance and the azimuthal angle variances are difficult to model due to the data generation process, the limitations of which were discussed above, and therefore, we use the standard maximum likelihood estimates calculated marginally from observed coordinates. On the other hand, the means 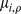, 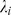 and 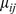 are central parameters representing a measure of the strength of the interaction between the fragment and the target.

#### 0.2.1 Parameter prior densities

A prior distribution in Bayesian statistics can either be used to model uncertainty about a parameter or to include *a priori* knowledge, or beliefs, in the model [21]. Both of these uses are necessary for our modeling purposes.

Prior densities can be chosen in various ways such that they are either conjugate distributions for a particular likelihood function, or some other probability densities possessing required statistical properties, such as an asymmetry. Priors utilized here for individual parameters in the model densities are summarized in Table 6. In Table 6, 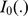 is the zeroth order modified Bessel function of the first kind. For the von Mises and Normal distributions, conjugate priors are used (for the mathematical derivations related to these distributions see [22], [23], [24]).

**Table 6:** Prior densities for model parameters.

Symbol	Parameter Type	Functional Form of Prior
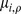	Mean of 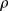 (i:th Normal component)	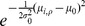 ;
		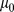 and 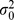 are constants.
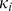	Concentration of 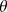 (i:th von Mises component)	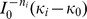 ;
		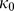 and 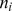 are constants.
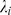	Mean of 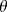 (i:th von Mises component)	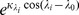 ;
		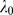 is a constant.
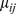	Mean of 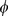 (j:th Normal component related to i:th von Mises component)	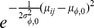 ; 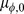 and
		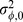 are constants.

Our prior density for the parameters is a slightly modified version of the distributions considered in [25] and [26]. The density has the form:
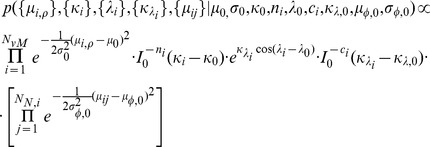
(8)


In equation (8) hyperparameters 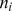 and 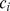 represent measures of modeler's belief in the expected values of the concentrations. The larger the value of the hyperparameter, the more the prior is concentrated around 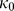 or 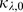. A default choice for any of these hyperparameters is the number of observations available for calculating the estimates for 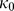 and 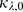.

#### 0.2.2 The posterior distribution

In Bayesian statistics, learning from observations takes place through the posterior distribution which is accessible from the joint probability density defined for the data and the parameters. For our piece-wise defined likelihood, the joint density is formally defined as
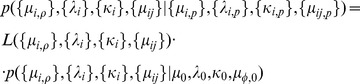
(9)


The posterior density (eq. 9) has the same functional form as the prior density (eq. 8), but with updated parameters. As shown in the equation (9), the prior parameters 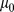, 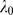, 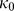 and 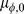 are updated to 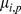, 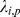, 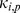 and 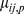, respectively, in the usual manner in Bayesian inference. In contrary, the remaining parameters are determined either directly from the data or given a suitable value based on chemical knowledge.

In order to define contact preferences, every fragment class and target class pair has to be specified with some characteristics. Maximum *a posteriori* (MAP) estimates are in this respect suitable when the associated densities are (piecewise) unimodal. The MAP estimates of the parameters are defined and updated with new data according to the formulae in Table 7. The prior parameter 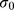 was given a constant value 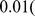
Å^2^) and 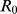 was defined separately for each fragment type.

**Table 7:** MAP estimates of model parameters.

Posterior variable	MAP estimate	Definitions and estimates
Mean of distance	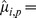	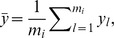
	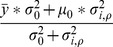	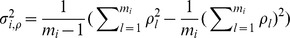
Mean of polar angle	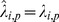	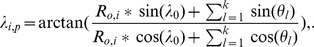
		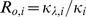 and 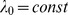
Concentration of polar angle	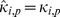	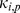 numerically by equalizing model and data variances.
Mean of azimuthal angle	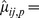	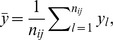
	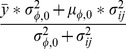	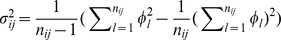

#### 0.2.3 Updated parameters and the probability mass in a reference volume

In our method, to evaluate the plausibility of a contact atom type in a given spatial area, the probability mass within in this volume is evaluated. The mass is calculated using the model densities (4) with updated parameters, see Table 7. The spatial area, or volume, is defined by a distance interval and a solid angle (i.e. intervals polar and azimuthal angles), and can be arbitrarily located. The volume that contains all target locations is defined through the intervals 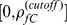, 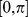 and 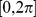 for the distance, polar angle and azimuthal angle, respectively. The cutoff 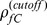
is the maximum distance used when collecting data for a fragment class and target class pair (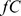). The size of the volume can be chosen to be large, when for example contact preferences on either side of the fragments plane are investigated. Alternatively, the size of the volume can also be small, depending on the situation under investigation.

The spatial information content of the model is coded in the particular functional form of the probability density, but if one would solely rely on probability densities, it could easily happen that a scarce contact atom type could get a hierarchically higher preference position than a relatively often encountered type. This could happen in a spatial area where all the few contacts of the former type are observed. These kinds of problems are avoided and results for different contact types made more directly comparable by supervising the model such that chemically more likely contacts are paralleled, as well as the less likely. The model supervision was here achieved by multiplying the probability masses with target type specific weights that are calculated from three parameters electronegativity, softness and mean distance. Softness of an element 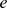, one from the group **G** = {C, N, O, S, F, CL, BR, I}, was defined as twice the mean value of hardness among the elements in **G**, minus hardness of the element 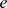. Numerical values for absolute hardness were taken from Parr et al. [27]. The electronegativity and softness were used to represent the tendency of an element to obtain partial charge in a compound. The third parameter, mean distance, is a measure of the strength of the interaction between a fragment and a target, and the numerical value given to it was the arithmetic mean of the distances in the training data. These parameters are used to calculate the weights as proportional to Coulomb force between the partial charges at the mean distance, i.e.
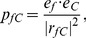
(10)where 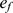 and 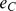 are the obtained partial charges for a fragment Main-atom and a target atom, respectively, and 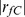 is the mean distance between the Main-atom and the target atom. The motivation for this prior is that the intermolecular interactions are mainly electrostatic, despite of the fact that they occur in many different forms, e.g., between a permanent dipole and an induced dipole, or between two induced dipoles, known as a London dispersion.

In the above formulation, it is assumed that a generic *a priori* information can be accurately utilized when modeling an interaction between 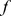 and 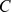. It would also be possible to use calculated energies of some simplified fragment-target model as the *a priori* information, but the described approach is chosen because of its simplicity and independence of molecular details, which follows from utilizing element specific, measurable parameters, i.e. ionization energy and electron affinity [27]. Calculated prior probabilities relevant for this study are given in Tables 8 and 9.

**Table 8:** Prior probabilities for fragment classes used in this study.

f 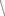 C	C3	C4	C5	C6	C7	C8	C9
f2	0.0015	0.0077	0.1259	0.0012	0.0015	0.0970	0.1355
f3	0.0014	0.0069	0.1217	0.0016	0.0014	0.0990	0.1312
f5	0.0027	0.1316	0.0876	0.0020	0.0020	0.1233	0.1246
f8	0.0018	0.0884	0.0896	0.0020	0.0006	0.1297	0.1317
f11	0.0103	0.0542	0.1248	0.0119	0.0120	0.0777	0.0301
f18	0.0013	0.1002	0.1000	0.0014	0.0014	0.1391	0.1237
f20	0.0109	0.0691	0.0698	0.0740	0.0713	0.0864	0.0863
f22	0.0068	0.0365	0.1052	0.0079	0.0080	0.0594	0.0229
f23	0.0058	0.0313	0.1016	0.0062	0.0066	0.0103	0.0187
f26	0.0020	0.0176	0.0966	0.0025	0.0022	0.0037	0.1563
f27	0.0023	0.0130	0.1083	0.0027	0.0025	0.0040	0.0656
f34	0.0112	0.0698	0.0712	0.0266	0.0739	0.0884	0.0871
f36	0.0110	0.0661	0.0729	0.0696	0.0835	0.0814	0.0779

Classes C3 to C9.

**Table 9:** Prior probabilities for fragment classes used in this study.

f 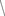 C	C10	C11	C12	C13	C14	C15
f2	0.1003	0.1036	0.1125	0.1100	0.1068	0.0960
f3	0.1073	0.1026	0.1092	0.1152	0.1090	0.0934
f5	0.1279	0.0036	0.1306	0.1384	0.0017	0.1238
f8	0.1351	0.0037	0.1347	0.1519	0.0019	0.1288
f11	0.0787	0.0894	0.1718	0.1716	0.0911	0.0763
f18	0.1380	0.0022	0.1366	0.1350	0.0012	0.1198
f20	0.0882	0.0891	0.0896	0.0894	0.0901	0.0858
f22	0.0641	0.1193	0.1938	0.1922	0.1200	0.0638
f23	0.0050	0.2090	0.2059	0.1926	0.2020	0.0049
f26	0.0018	0.1822	0.1815	0.1749	0.1768	0.0020
f27	0.0018	0.2025	0.2013	0.1939	0.2002	0.0018
f34	0.0890	0.0955	0.1011	0.1025	0.0991	0.0846
f36	0.0841	0.0890	0.0871	0.1069	0.0903	0.0803

Classes C10 to C15.

### 0.3 Hierarchy calculations

**Figure 3:**
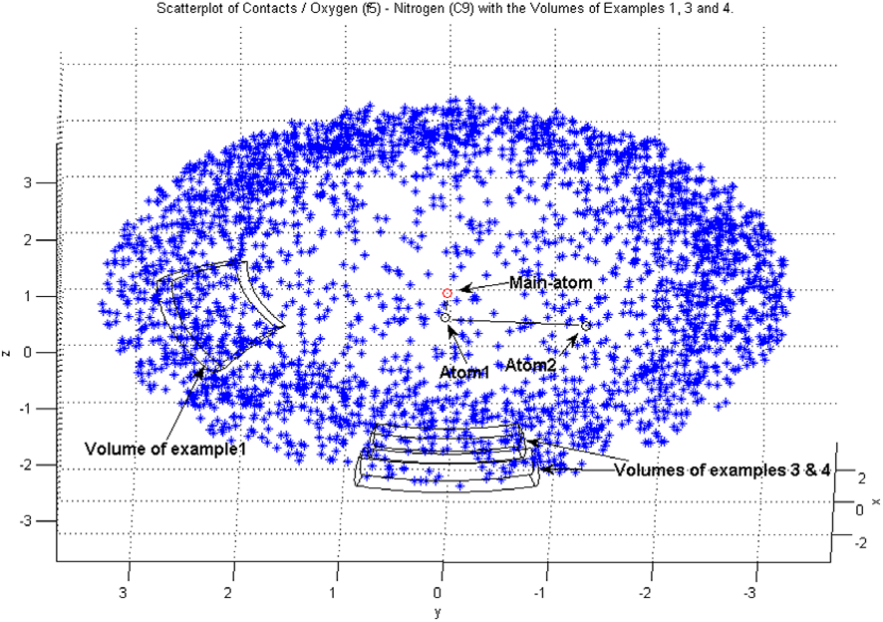
The volumes used for calculations in Examples 1,3 and 4. Here 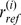 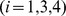 would be located in the center of the volume, both radially and with respect to the solid angle. The center of the volume of Example 2 (not shown explicitly) is located on the axis defined by the vector connecting Atom1 to Main-atom, i.e. on the positive x-axis in this figure.

In order to calculate the spatially dependent hierarchies around an arbitrary reference point
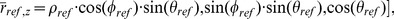
(11)we defined intervals in spherical polar coordinates:
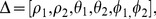
(12)which define a volume that includes 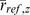, e.g. 
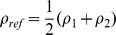
, 
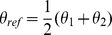
 and 
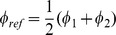
. The reference point 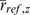 is defined in the reference frame that is used for modeling the data, and in which the fragment is in the (-z)(-x)-plane. The Main-atom (see section Data collection and processing) is at the origin, Atom1 on the negative z-axis and Atom2 in a point 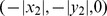, i.e. in the plane defined by the negative z- and x-axes. On the other hand, in the reference frame used for the graphical representations in this article, 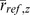 (eq. 11) is transformed to
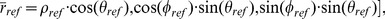
(13)which is in a reference frame where the fragment is in (-x)(-y)-plane, see Figures 3 and 1.

The probability masses in the volume defined by 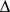 (eq. 12) are evaluated using the model densities with the updated parameter values. Technically the calculations are done either with series expansions, see e.g. the equations 7.1.1., 7.1.7., 7.1.22 and 9.6.34 in [28] or directly as Riemann sums.

The 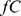-specific probability mass is the factor that gives a contact atom type 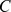 its rank, in the fragment class 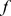 related, and around a reference point 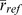 defined hierarchy. Namely, the bigger the mass, the more probable the contact atom type. The volume can cover a larger portion of the neighborhood of the fragment, for example, the hemisphere on either side of the plane of the fragment.

A hierarchy can also be defined for example among the fragment types (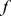), with respect to a representative of a target class 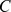 around 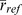. The motivation for choosing the probability density and the estimation procedures of the model parameters as described in Methods, is that they provide a rapid and flexible way to capture the relevant features of the target atom distributions, without relying on fine details of the target atom clouds, which are potentially misleading due to the intrinsic uncertainties in the data generation process.

## Results

The functionality of the introduced Bayesian method of finding hierarchies is here illustrated by a number of case-studies. The calculated hierarchies are compared with contact atom type counts found in the reference data, in the volume surrounding the reference point 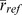, representing a target atom location. The hierarchies and the reference frequencies are not expected to be perfectly congruent, due to the fact that the reference data set is not exhaustively large. Therefore, they are expected to simply show some of the specific features around the reference point 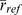. In order to illustrate the reliability of the results, standard errors for all results were determined with a bootstrap type procedure [29], which is described together with the corresponding results.

### Example 1: A typical location of a hydrogen bonding partner

**Figure 4:**
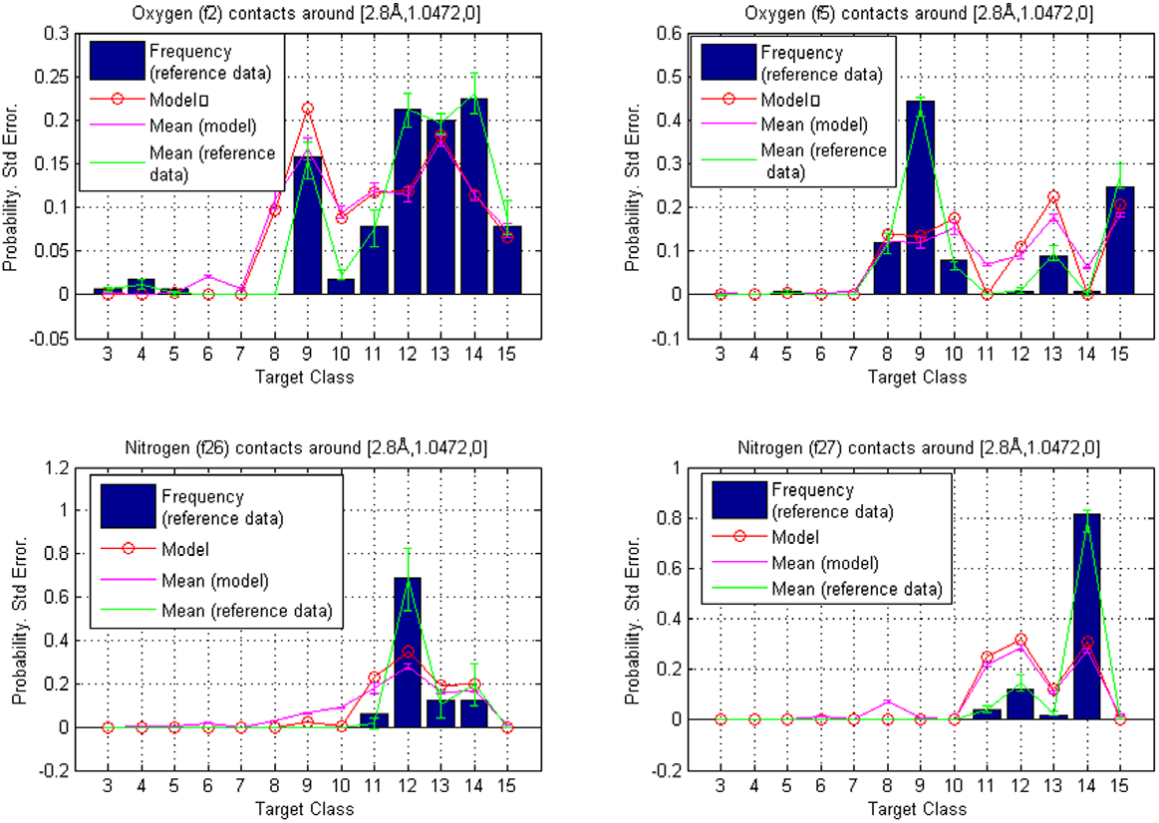
Reference point 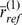
 **.** Comparison of contact atom frequencies with model based probabilities for fragment classes **f2**, **f5**, **f26** and **f27**. Hierarchy is given by the calculated probabilities, which are represented as circles. The circles are joined with a line to illustrate tendencies among target classes. The bars represent the fractions of target atoms belonging to a particular class. Both sum to one over target classes. Also shown are standard errors for both the frequencies and the model based probabilities (joined with lines and centered around the mean values). The contact atom counts in reference data for **f2**, **f5**, **f26** and **f27** were 165, 126, 16 and 49, respectively.

First, we consider a reference point equal to
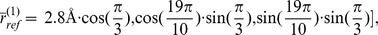
which is located just below the plane of the fragment, with a polar angle deviation of 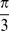 to the left from the main direction, see Figure 3. The intervals 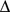 (eq. 12) used were
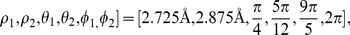
and the results calculated around 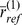 for fragment classes **f2**, **f5**, **f8**, **f11**, **f18**, **f22**, **f23** and **f27** (see Table 2) are given in Table 10 below. They are also represented graphically for **f2**, **f5**, **f26** and **f27** in Figure 4. The error bars represent separately standard errors for the model based probabilities and the frequencies in reference data. They were defined by calculating both quantities in a set of 1,000 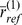 centered volumes, that were slightly different in shape and size. To investigate how the inclusion of additives as ligands in the training and reference data sets would affect the model-based rankings, we divided the data into two sets using a list of approximately 770 additives that are found in PDB [30]. Contact data for these additives was removed from the original set to create a new additive-free set. Then, based on the model trained with the new dataset, contact hierarchies were calculated and probability masses were compared with reference data that also lacked the additives.

**Table 10:** Model based probabilities calculated from the reference point 1 centered volume.

f 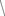 C	f2	f5	f8	f11	f18	f22	f23	f27
C3	0.0000	0.0001	0.0000	0.0000	0.0006	0.0000	0.0000	0.0000
C4	0.0000	0.0003	0.0000	0.0000	0.0070	0.0000	0.0000	0.0000
C5	0.0024	0.0038	0.0019	0.0000	0.0178	0.0000	0.0000	0.0000
C6	0.0003	0.0001	0.0000	0.0317	0.0000	0.0006	0.0001	0.0002
C7	0.0001	0.0001	0.0001	0.0104	0.0000	0.0000	0.0004	0.0000
C8	0.0976	0.1382	0.1734	0.0675	0.2759	0.0115	0.0049	0.0016
C9	0.2139	0.1342	0.1531	0.0014	0.0867	0.0015	0.0004	0.0023
C10	0.0870	0.1760	0.1706	0.0684	0.3998	0.0005	0.0000	0.0000
C11	0.1179	0.0022	0.0027	0.0370	0.0001	0.5804	0.1848	0.2506
C12	0.1191	0.1105	0.1078	0.0967	0.0297	0.0865	0.4721	0.3196
C13	0.1823	0.2259	0.1851	0.5027	0.0937	0.1331	0.0837	0.1186
C14	0.1141	0.0010	0.0014	0.0165	0.0008	0.1601	0.2533	0.3070
C15	0.0653	0.2075	0.2039	0.1677	0.0876	0.0257	0.0002	0.0002

**Figure 5:**
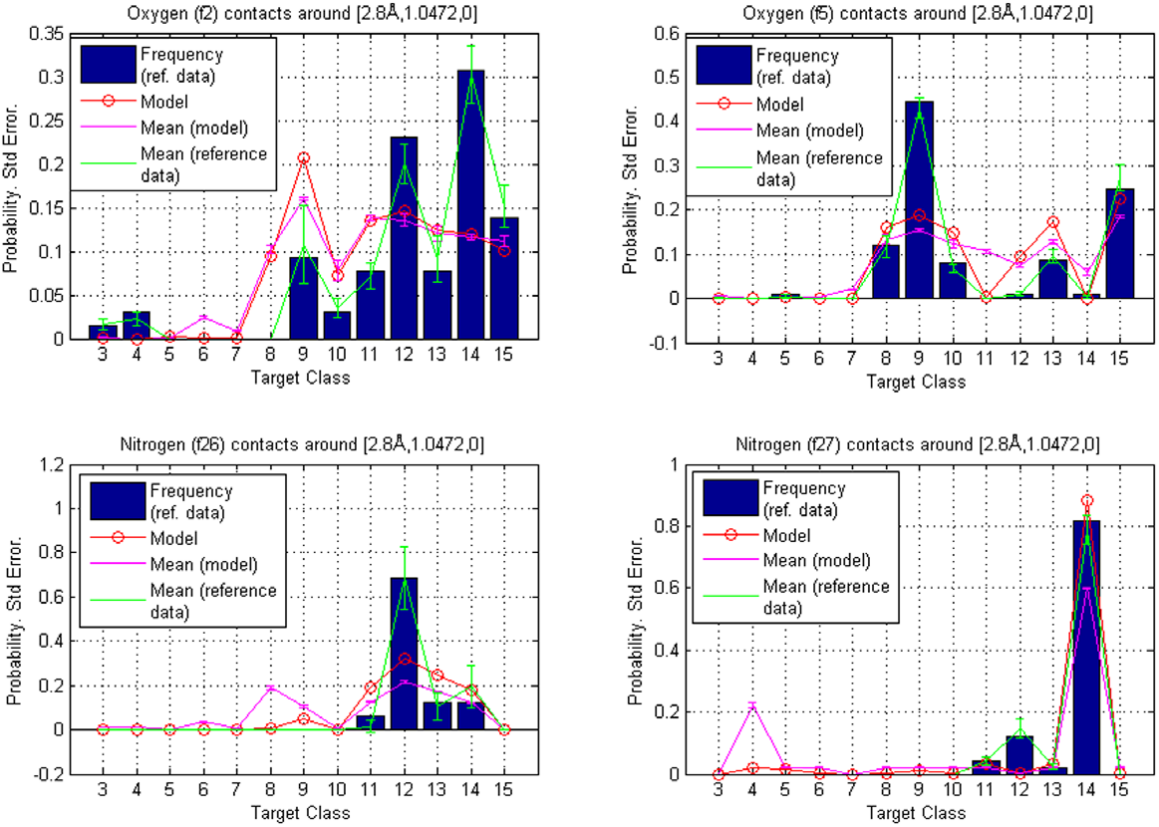
Hierarchies from model trained with data that excludes the additive-type ligands. Reference point 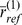: comparison of contact atom frequencies with model based probabilities for fragment classes **f2**, **f5**, **f26** and **f27**. Hierarchy is given by the calculated probabilities, which are represented as circles. The circles are joined with a line to illustrate tendencies among target classes. The bars represent the fractions of target atoms belonging to a particular class. Both sum to one over target classes. Also shown are standard errors for both the frequencies and the model based probabilities (joined with lines and centered around the mean values). The contact atom counts in reference data for **f2**, **f5**, **f26** and **f27** were 65, 126, 16 and 49, respectively.

**Figure 6:**
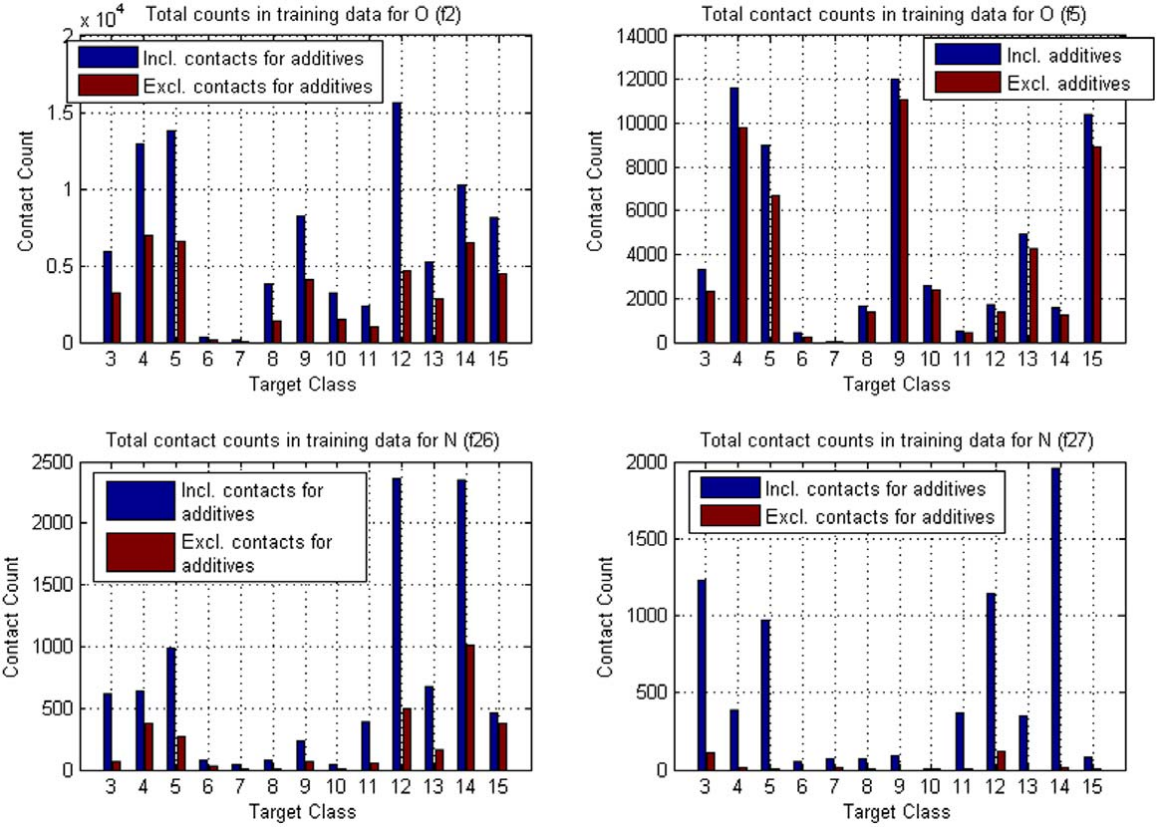
Target class specific total counts of contacts. Fragment classes **f2**, **f5**, **f26** and **f27**, the same as in Example 1 related hierarchy figure.

We replicated the calculations in Example 1 using the additive-free data and the results are shown in Figure 5 and Table 11. In addition, a comparison of the original and new contact atom counts is presented graphically in Figure 6. Comparisons of the rankings reveal that they are nearly identical in the two situations. Nevertheless, some discrepancy does occur and this example illustrates the need of a careful consideration about what structures can be included as representative information to the training data. If predictions based on multiple different training sets are extensively monitored and discovered to be sufficiently similar, it is possible to consider the use of a universal training data collection where the different sets are merged.

**Table 11:** Model based probabilities calculated from the reference poin 1 centerd volume.

f 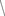 C	f2	f5	f8	f11	f18	f22	f23	f27
C3	0.0000	0.0001	0.0001	0.0118	0.0012	0.0000	0.0008	0.0000
C4	0.0000	0.0000	0.0004	0.0619	0.1062	0.0000	0.0001	0.0214
C5	0.0016	0.0034	0.0023	0.0000	0.0616	0.0000	0.0003	0.0174
C6	0.0004	0.0001	0.0001	0.0136	0.0005	0.0000	0.0000	0.0004
C7	0.0001	0.0004	0.0000	0.0137	0.0005	0.0002	0.0006	0.0000
C8	0.0939	0.1610	0.1511	0.0888	0.0534	0.0016	0.0000	0.0006
C9	0.2071	0.1891	0.2407	0.0344	0.2019	0.0075	0.0001	0.0105
C10	0.0725	0.1497	0.1611	0.0900	0.0530	0.0017	0.0000	0.0003
C11	0.1352	0.0037	0.0021	0.1022	0.0009	0.0376	0.9775	0.0325
C12	0.1459	0.0951	0.1023	0.1963	0.3106	0.5200	0.0009	0.0023
C13	0.1243	0.1722	0.1491	0.1961	0.0991	0.2575	0.0008	0.0311
C14	0.1188	0.0010	0.0017	0.1041	0.0020	0.1722	0.0193	0.8830
C15	0.1003	0.2243	0.1890	0.0872	0.1091	0.0017	0.0000	0.0003

The probability masses have been obtained with a model that was trained using a dataset from which the additives were excluded.

### Example 2: A possible halogen bond geometry

The second reference point is
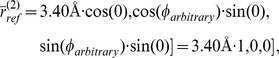
i.e. on the positive x-axis when defined in the reference frame of Figure 3. The intervals 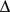 (see eq. 12) used were
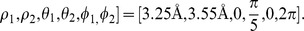

**Figure 7:**
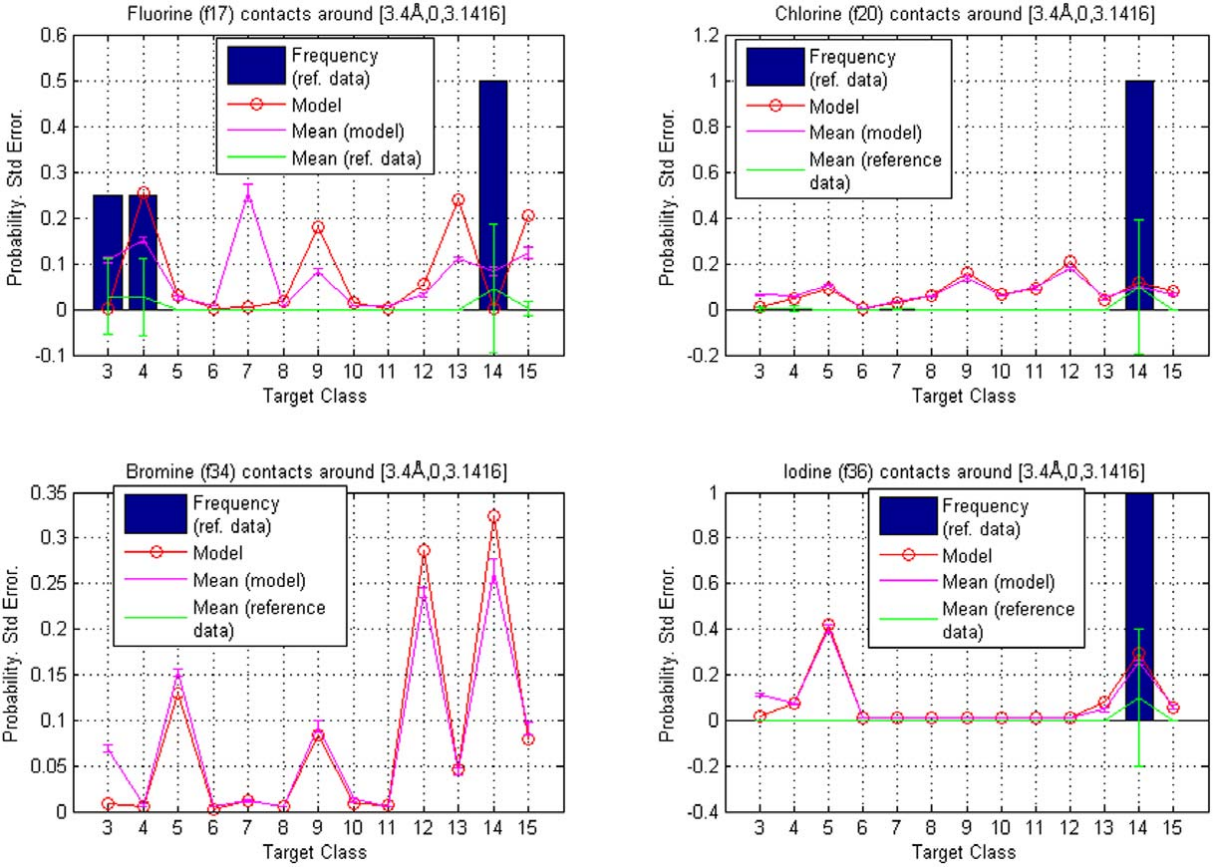
Reference point 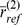
 **.** Comparison of contact atom frequencies with model based probabilities for fragment classes **f17**, **f20**, **f34** and **f36**. Hierarchy is given by the calculated probabilities, which are represented as circles. The circles are joined with a line to illustrate tendencies among target classes. The bars represent the fractions of target atoms belonging to a particular class. Both sum to one over target classes. Also shown are standard errors for both the frequencies and the model based probabilities (joined with lines and centered around the mean values). The contact atom counts in reference data for **f17**, **f20**, **f34** and **f36** are 4, 7, 0 and 1, respectively.

Table 12 presents probabilities for classes **f13**, **f17**, **f18**, **f20**, **f21**, **f34**, **f35** and **f36** and the results are represented graphically for **f17**, **f20**, **f34** and **f36** in Figure 7. The error bars represent separately standard errors for the model based probabilities and the frequencies in reference data. As in the previous example, they were defined by calculating both in a set of 1,000 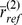 centered volumes, that were slightly different in shape and size.

**Table 12:** Model based probabilities calculated from the reference point 2 centered volume.

C 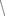 f	f13	f17	f18	f20	f21	f34	f35	f36
C3	0.0061	0.0024	0.0026	0.0083	0.0170	0.0083	0.0416	0.0153
C4	0.0938	0.2534	0.2609	0.0462	0.0157	0.0055	0.0094	0.0724
C5	0.1470	0.0295	0.0595	0.0935	0.1158	0.1301	0.5172	0.4147
C6	0.0017	0.0002	0.0039	0.0058	0.0061	0.0018	0.0102	0.0082
C7	0.0017	0.0057	0.0041	0.0281	0.0049	0.0120	0.0093	0.0098
C8	0.0069	0.0157	0.1322	0.0569	0.3050	0.0058	0.0140	0.0096
C9	0.0152	0.1801	0.1610	0.1620	0.0102	0.0845	0.0101	0.0091
C10	0.0011	0.0140	0.0996	0.0664	0.0070	0.0092	0.0148	0.0099
C11	0.2086	0.0003	0.0100	0.0890	0.0000	0.0063	0.0120	0.0105
C12	0.2357	0.0557	0.0998	0.2086	0.0907	0.2868	0.0143	0.0102
C13	0.0683	0.2377	0.0142	0.0430	0.1396	0.0457	0.0123	0.0825
C14	0.2126	0.0013	0.0023	0.1138	0.1905	0.3247	0.3239	0.2918
C15	0.0011	0.2037	0.1499	0.0783	0.0975	0.0793	0.0109	0.0533

### Examples 3 and 4: Two distances in a direction perpendicular to the plane of the fragment

The third and fourth reference points, in the reference frame of Figure 3, are
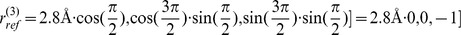


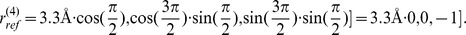



**Figure 8:**
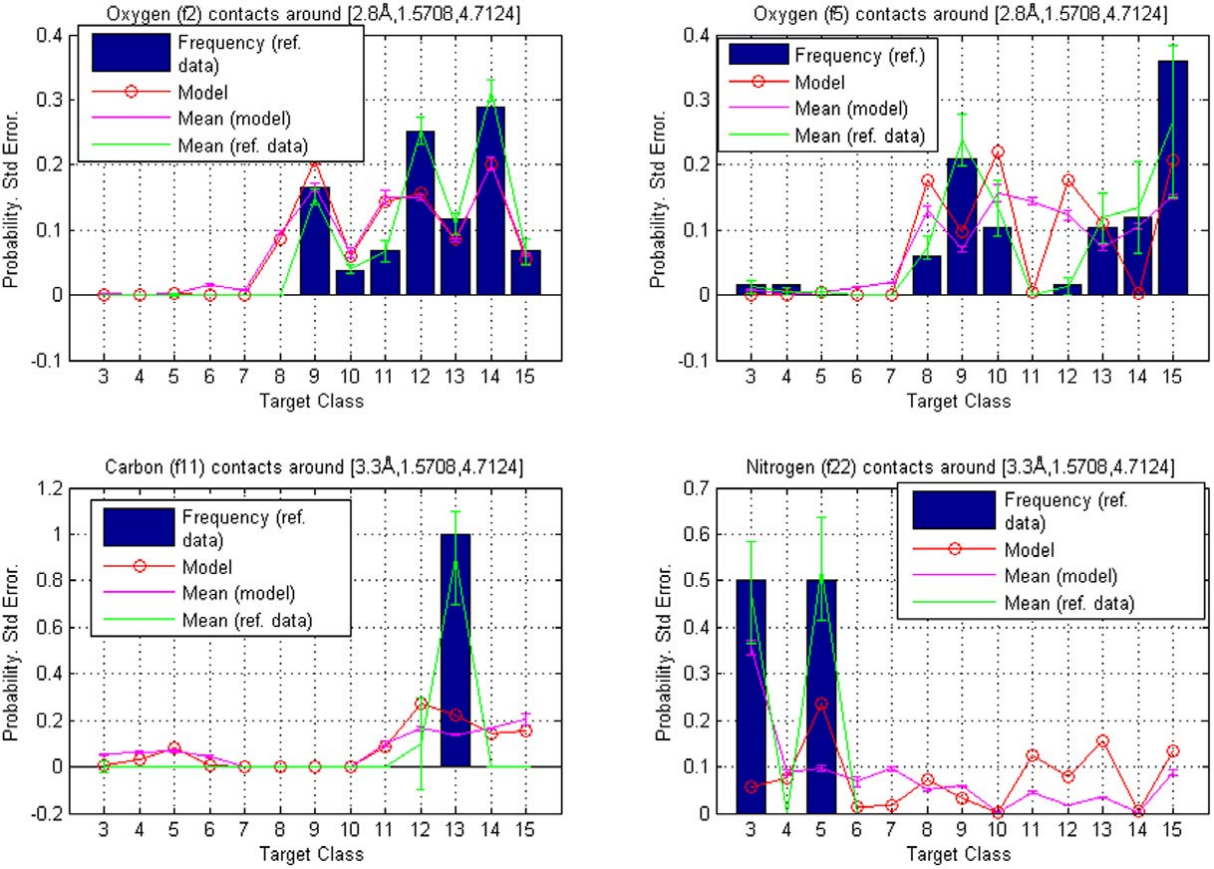
Reference points 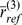
 **and** 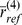 **.** Comparison of contact atom frequencies with model based probabilities for fragment classes **f2**, **f5**, **f11** and **f22**. Hierarchy is given by the calculated probabilities, which are represented as circles. The circles are joined with a line to illustrate tendencies among target classes. The bars represent the fractions of target atoms belonging to a particular class. Both sum to one over target classes. Also shown are standard errors for both the frequencies and the model based probabilities (joined with lines and centered around the mean values). The contact atom counts in reference data for **f2**, **f5**, **f11** and **f22** are 158, 163, 5 and 7, respectively.

The intervals 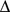 (see eq. 12) used were
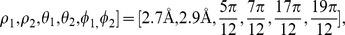


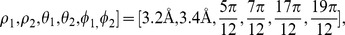
and the corresponding volumes are centered around the negative z-axis, as shown in Figure 3. The results are represented graphically for fragment classes **f2**, **f5**, **f11** and **f22** in Figure 8. The error bars represent separately standard errors for the model based probabilities and the frequencies in reference data, calculated in the same manner as in the previous examples.

**Figure 9:**
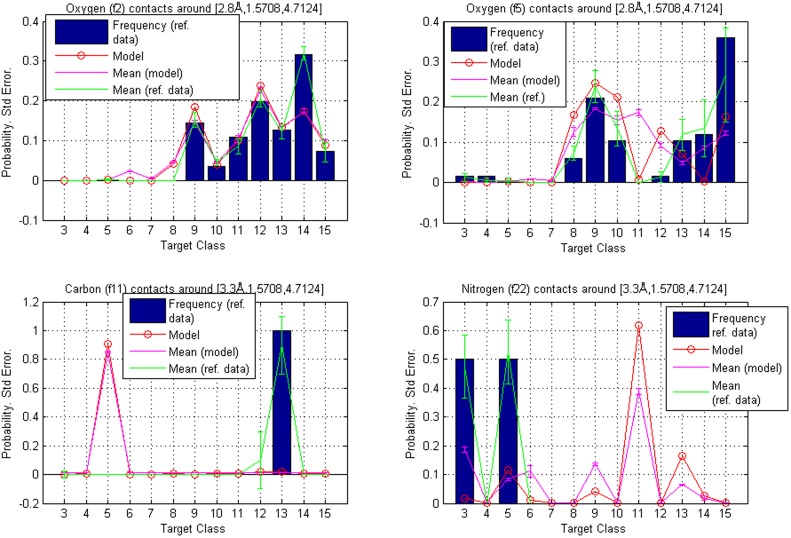
Hierarchies from model trained with data that excludes the additive-type ligands. These are relating to example 1. Reference points 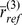 and 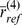: comparison of contact atom frequencies with model based probabilities for fragment classes **f2**, **f5**, **f11** and **f22**. Hierarchy is given by the calculated probabilities, which are represented as circles. The circles are joined with a line to illustrate tendencies among target classes. The bars represent the fractions of target atoms belonging to a particular class. Both sum to one over target classes. Also shown are standard errors for both the frequencies and the model based probabilities (joined with lines and centered around the mean values). The contact atom counts in reference data for **f2**, **f5**, **f11** and **f22** are 111, 67, 1 and 6, respectively.

**Figure 10:**
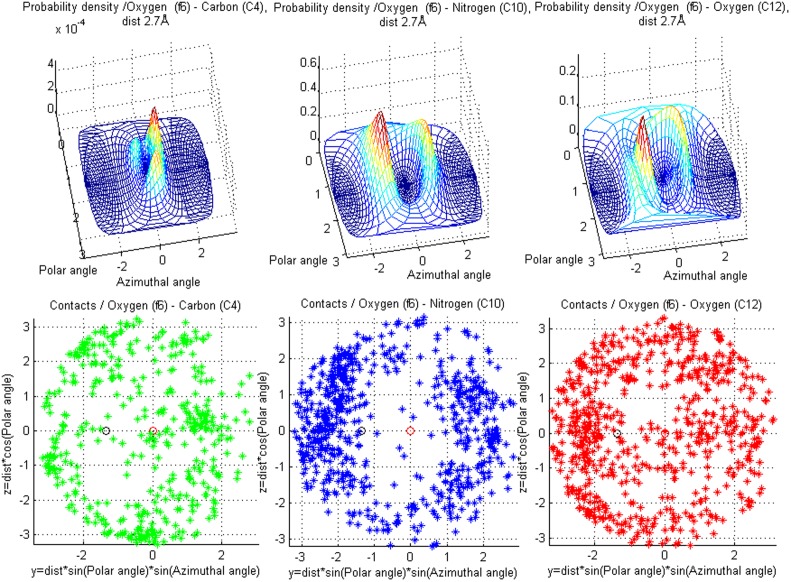
Contacts for carboxyl oxygen (f6). Showing differences in spatial arrangement of contacts among three target classes - **C4** (alpha carbon), **C10** (amino nitrogen) and **C12** (carboxyl oxygen). The scatter plots contain all the target atoms found in the fragment class **f6** training data for these target classes.

**Figure 11:**
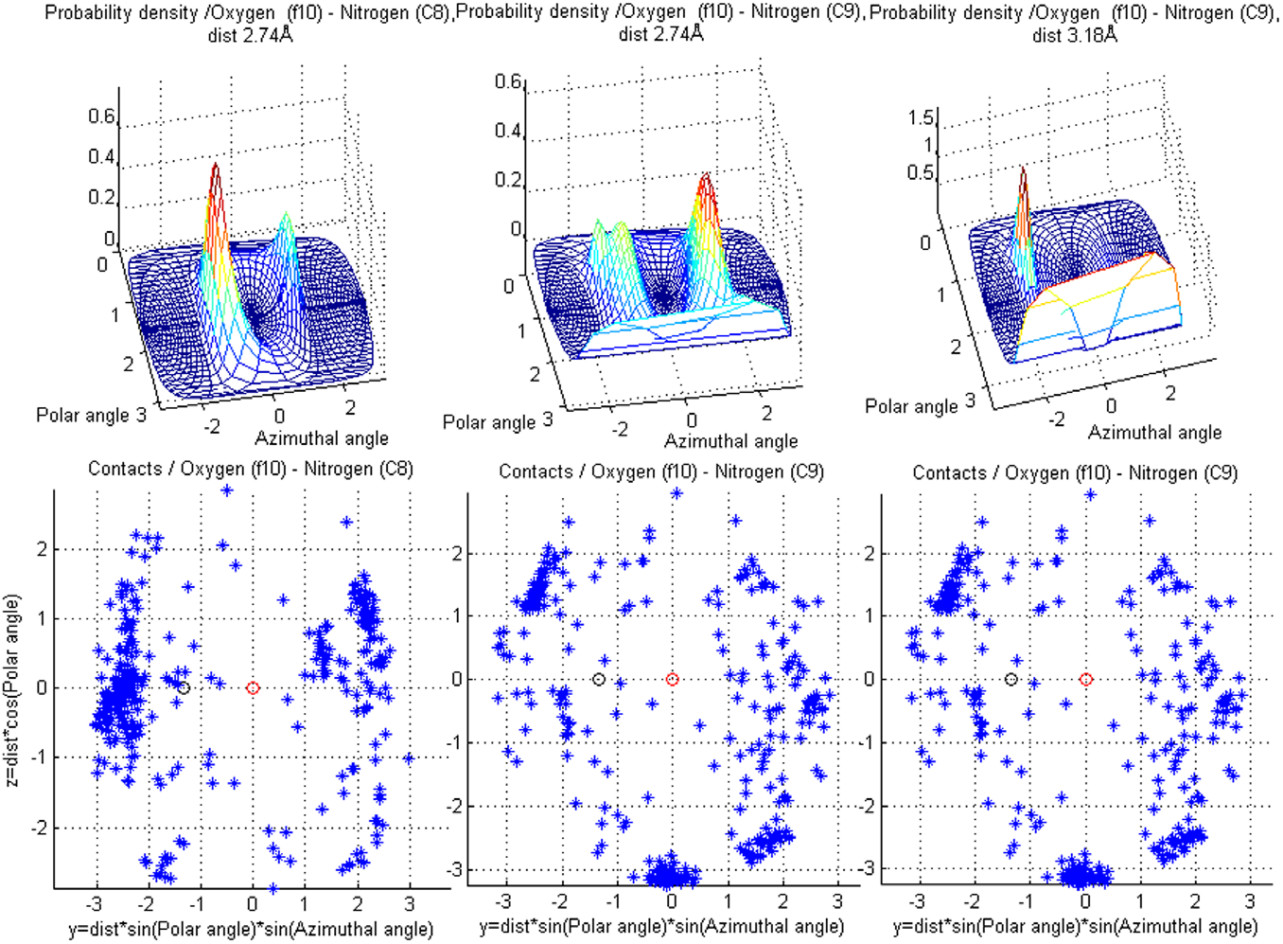
Contacts for amide oxygen (f10). Showing differences in contact arrangements for two target classes - **C8** (carbamoyl nitrogen) and **C9** (imidazole, guanido or indole nitrogen), and also distance dependence for class **C9**. The scatter plots contain all contacts found in the training data for these fragment and target class pairs. Note that the target atom clouds in the middle and on the right are the same.

**Figure 12:**
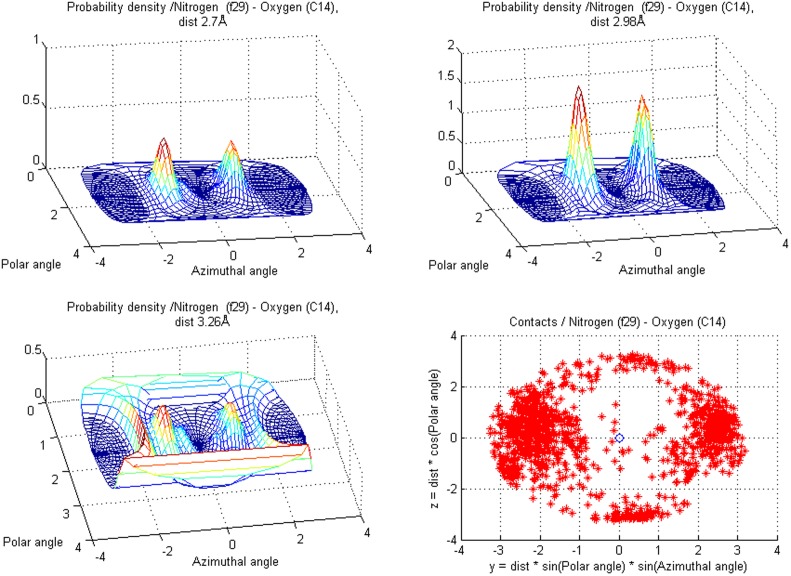
Contacts for nitrogen, singly bonded to a planar structure (f29) — e.g. in carbamoyl group. showing distance dependence for target class **C14** (carbonyl oxygen). The scatter plot contains all **C14** target atoms in the training data and the densities show which directions are emphasized at distances 2.7, 2.98 and 3.26 Å.

Hierarchies calculated when the additives were excluded, as discussed in Example 1, are presented in Figure 9. It is seen that a larger discrepancy in the model-based rankings compared with the reference data occurs when the reference data are extremely sparse, which makes the unsmoothed relative frequencies highly volatile. To also visualize more generally how our method smooths scatter data about different types of contacts, 3D-plots of estimated densities are shown in Figures 10, 11, 12.

### Example 5: Direct contacts of R-norepinephrine

Here we consider the hydrogen bonding and aromatic interaction preferences of norepinephrine (also known as noradrenaline; PDB ligand identifier: LT4). The molecular environment of this example is the norepinephrine binding site in chain B of human phenylethanolamine N-methyltransferase (PNMT) from PDB entry 3HCD. PNMT catalyses adrenaline synthesis with coenzyme S-adenosyl-L-methionine (AdoMet). In the structure of 3HCD AdoMet is replaced by its demethylated form S-adenosyl-L-homocysteine (AdoHcy) to study the binding mode of LT4 [31]. The x-ray resolution of the entry 3HCD is 2.39 Å , which is near the upper limit considered in our study (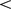
2.5 Å , see Table 1). As discussed previously, this means that some precaution is necessary while deducing interactions from the structure. Consequently, the statistical nature of our method is helpful, since the probability densities can indicate a certain relative location that is associated with what is experimentally observed in other structures. This enable the quantification of the relation in question as a probability.

LT4 has three hydroxyl groups, a terminal amino group and a six-carbon aromatic ring as its functional groups. The most preferred target class for any of these functional groups is defined as a class for which the product of the probability density peak value (eq. 3) and the class conditional prior probability (see Tables 8 and 9) has the highest numerical value.

Two of the hydroxyl oxygens are bonded to the aromatic ring (cathecol hydroxyl groups), and based on the model, prefer a nitrogen from histidine or arginine side chain as a contact. The aromatic ring carbons then prefer aromatic carbons, i.e. phenylalanine, histidine, tyrosine or tryptophan is a likely contact residue. The aromatic carbons also have strong contacts from the hydrophilic targets, for example carboxyl oxygens. The hydroxyl group of the aliphatic tail prefers lysine and the terminal amino group prefers glutamic acid/glutamate or aspartic acid/aspartate.

**Figure 13:**
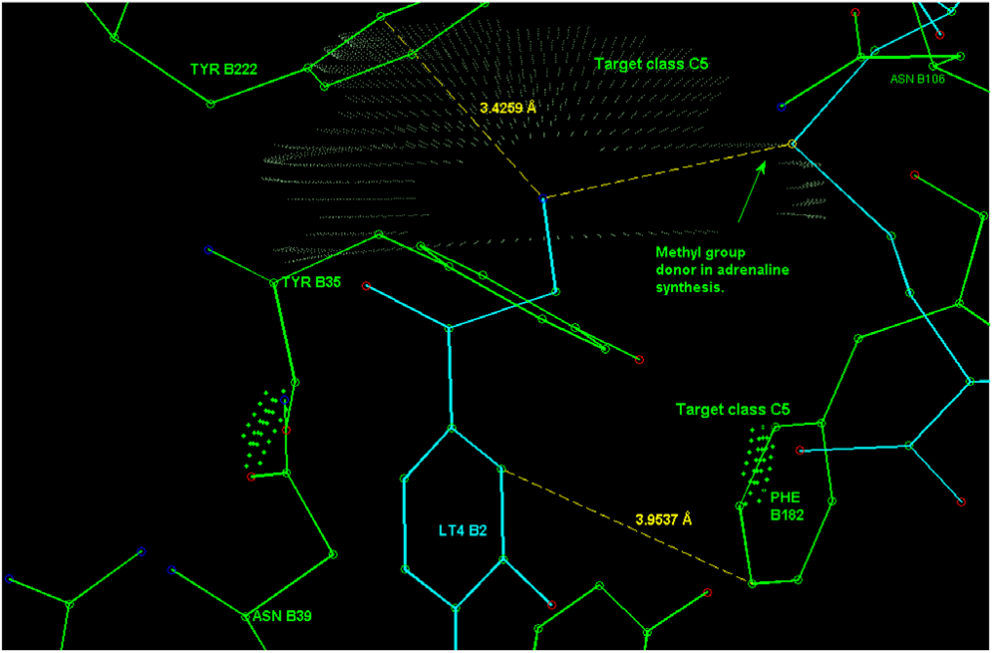
The aromatic phenylalanine contact PHE B182 in PNMT (see text of section Example 4) for the LT4 aromatic ring. Also depicted in the figure is the proximity (closest 3.4 Å ) of the TYR B222 aromatic ring to the amino group of LT4. Though the distance and orientation of the ring fit well to the hydrogen bond donor - aromatic ring interaction scheme, the relative direction is such that TYR B222 corresponds to probability density values smaller than 20% of the peak value. Therefore, this might not be a strong contact for the amino group, but possibly has a guiding task in the binding process. The depicted distance between amino group and the methyl group donating sulfur atom is 5.66 Å.

**Figure 14:**
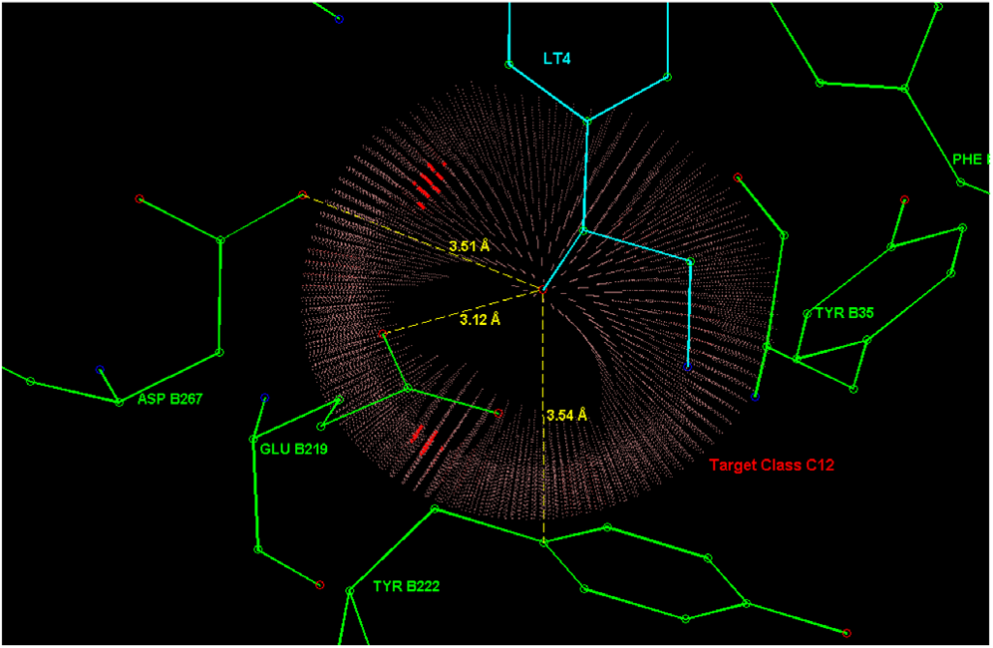
Contacts in the active site of PNMT (a methyltransferase) for R-noradrenaline tail hydroxyl group. Three amino acid residues (ASP B267, GLU B219 and TYR B222) were considered as contacts.

**Figure 15:**
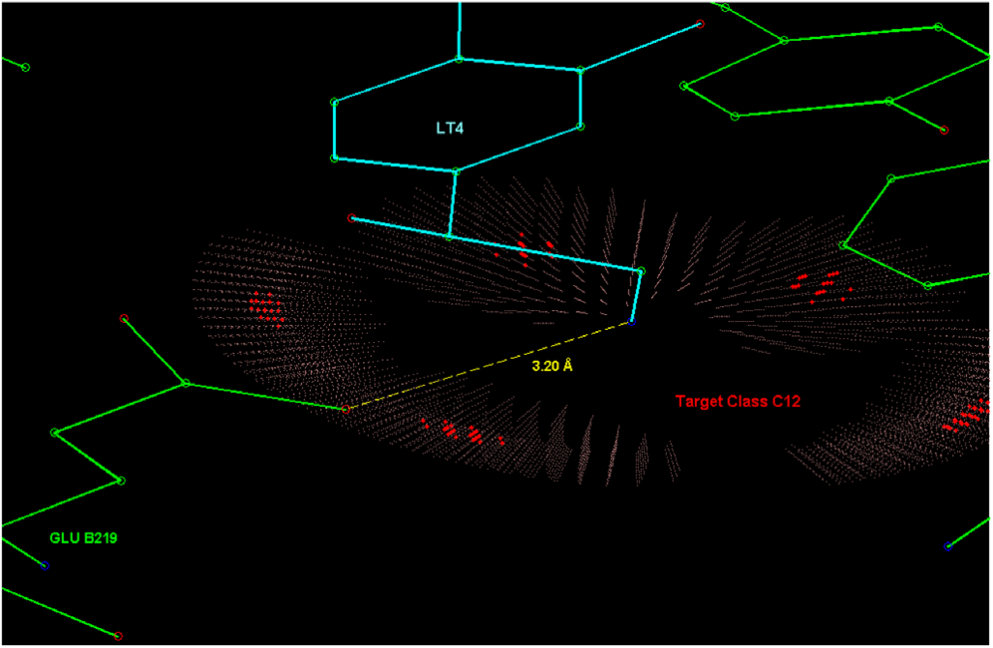
Carboxyl oxygen (C12) contact for the amino group of LT4. The residue GLU B219 carboxyl oxygen is located in a typical direction of a class C12 hydrogen bond acceptor for this functional group (fragment class **f26**).

**Figure 16:**
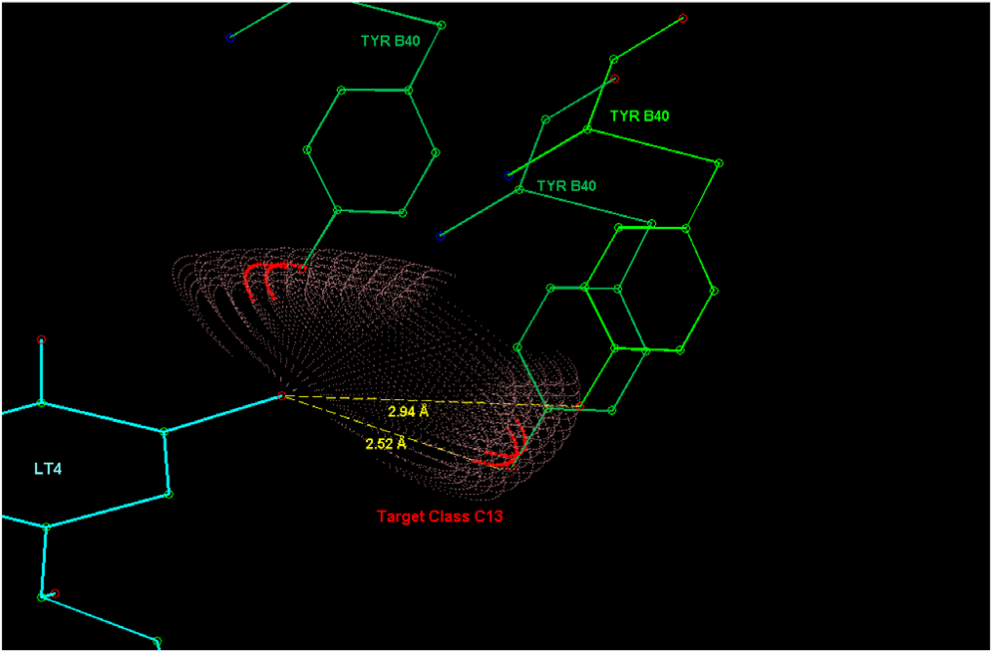
The contact between a cathecol hydroxyl group and the tyrosine B40 residue of the phenylethanolamine N-methyltransferase (PNMT). The distance to both degenerate maxima is 2.52 Å.

**Figure 17:**
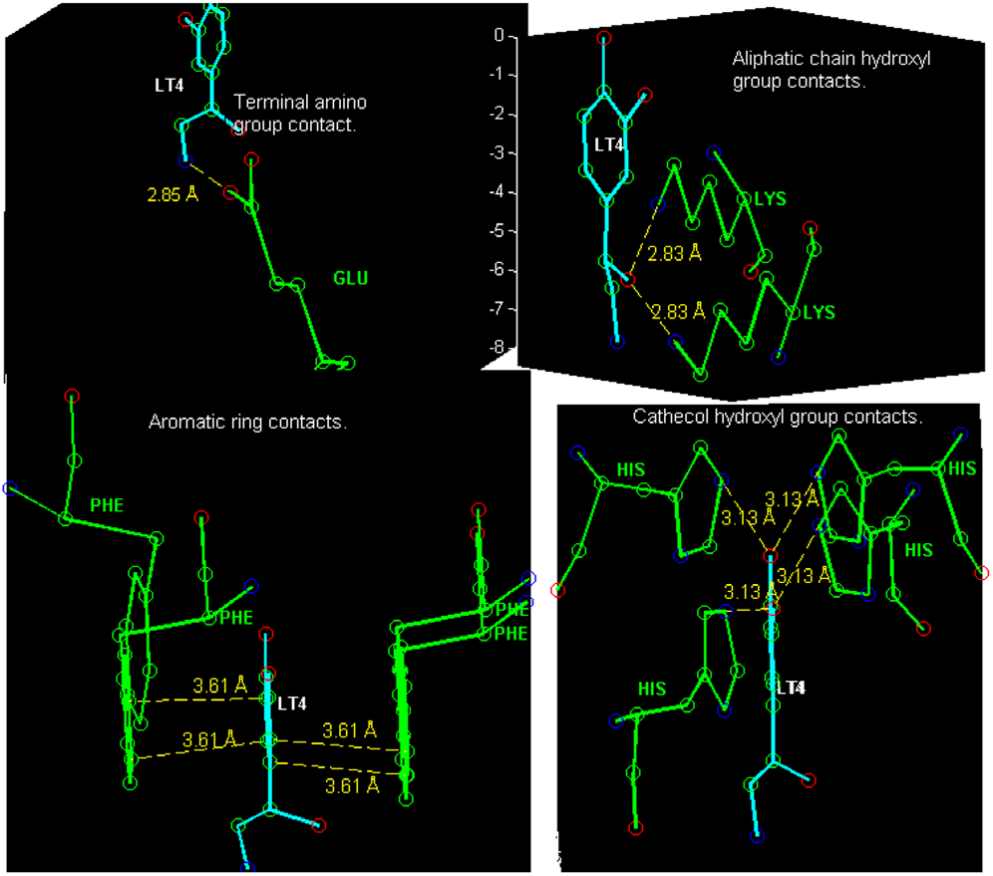
R-Norepinephrine (PDB ligand identifier: LT4) with amino acid residues that contain target atoms having highest probability density values in the model. In the figures on the right, the double contacts are created by a degeneracy that follows from the way the fragments are defined. Namely, the third atom (Atom2, see Section Data collection and processing) of a fragment in a molecule can frequently be chosen from more than one possible atom and each choice creates its own probability density, including a maximum. These densities are connected through a rotation around the covalent bond between the Main-atom and Atom1.

These *a priori* preferences are not related to LT4, instead only the 3D structure of the interactions is. Some directional aspects related to this example are illustrated in Figures 13, 14, 15, 16, 17. Figures 14 and 15 present hydrogen bonding contacts for the R-norepinephrine tail. There are two carboxyl oxygen (**C12**) contacts for the LT4 hydroxyl group, namely GLU B219 and ASP B267. The former is at a distance less than the maximum length used in this study for a oxygen donor and oxygen acceptor hydrogen bond, i.e. 3.14 Å
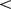
3.30 Å . According to the model, its direction of approach is not typical for a hydrogen bond, but because the same GLU B219 residue is simultaneously a contact for the adjacent amino group, the actual preferred direction is such that it allows the carboxyl to bond with both of these functional groups in LT4. Therefore this is considered a direct contact. Regarding the amino group, the direction of approach of the GLU B219 carboxyl oxygen is typical for a hydrogen bond (almost optimal), only somewhat shifted to a direction that facilitates the double contact described above, see Figure 15. The latter carboxyl, ASP B267, is in a more typical direction, but even further apart, and it is confirmed from PDB entry 3HCD water locations that this contact is a bridged hydrogen bond, not a direct contact.

The TYR B222 contact for the hydroxyl of LT4 tail, see Figure 14, has an aromatic ring that can serve as a hydrogen bond acceptor. Therefore, in case the LT4 tail hydroxyl group would act as an acceptor in the above described hydrogen bonds (i.e. with water and GLU B219) it could in principle donate its hydrogen to a weak hydrogen bond with the aromatic ring of TYR B222, because the closest atom of the ring is at a distance of about 3.5 Å and the ring is facing toward the hydroxyl group. Consequently, as for the LT4 amino group this is not a strong contact, but perhaps has a guiding task in the binding process.

### Example 6: Separating COMT ligands from decoys in a subset of DUD

Here we demonstrate the usefulness of the estimated contact probability masses in discriminating between appropriate and poor binders using logistic regression, see e.g. [32]. In the current application context, logistic regression model connects a binary response variable (here ligand/decoy), with explanatory variables describing the modeled system. The outcome is a probability indicating how likely it is for a system to belong to either of the two response groups. Our testing was done by retrieving a set containing 6 out of the 11 Catechol-O-methyltransferase (COMT) ligands and 19 out of the total 468 decoys from the Directory of useful decoys (DUD) [33]. Two extra ligands were added, namely dopamine and BIA 3-335 (PDB Ligand identifiers LDP and BIA, respectively), by retrieving their structures from ZINC database, see [34]. These 27 small molecules (ZINC codes in Tables 13, 14 and 15) were chosen so that the DUD molecules have high mutual resemblance, especially so that the decoys have an aromatic ring with at least two primary oxygens bonded to neighboring carbons (in hydroxyl groups typically). This is because all except one COMT ligand in DUD have this type of a structure, and the exception is different only in that the ring is non-aromatic. The two extra ligands were included for reference, because they are known good binders, for BIA [35], and should have clearly higher preference to binding than an average decoy.

**Table 13:** Ligand properties in Example 6.

Index	ZINC	pmPerMass	RotBonds	Phob/Phil
1	21789	0.053	2	2.33
2	330141	0.071	0	6
3	3801154	0.038	3	6
4	3814483	0.011	2	2
5	3814484	0.049	1	6
6	3814485	0.051	1	6
7	33882	0.052	2	6
8	52627624	0.078	5	12

Index values represent an ordering of the molecules used in this study.

**Table 14:** Properties of the first set of decoys in Example 6.

Index	ZINC	pmPerMass	RotBonds	Phob/Phil
9	22831	0.049	0	3
10	366295	0.050	3	2
11	366296	0.044	3	2
12	370041	0.033	3	2.5
13	370042	0.036	3	2.4
14	370157	0.015	2	4.5
15	370162	0.029	2	4.5
16	402870	0.055	2	3
17	438536	0.032	2	3
18	1833085	0.010	1	3.67

Index values represent an ordering of the molecules used in this study.

**Table 15:** Properties of the second set of decoys in Example 6.

Index	ZINC	pmPerMass	RotBonds	Phob/Phil
19	2519115	0.050	2	4.5
20	2990158	0.010	1	3.33
21	3836392	0.000	2	3
22	3871444	0.041	3	4.5
23	3973802	0.000	2	3
24	3995296	0.040	3	2
25	4000727	0.030	3	2
26	4404113	0.036	1	2.33
27	4443675	0.039	3	4.5

Index values represent an ordering of the molecules in this study.

The search for the binding mode of the small molecule in the binding pocket (from PDB ID 1H1D) was started by orienting the molecule such that two of the primary oxygens would coordinate with the magnesium ion (Mg^2+^), participating in the COMT function (see [35]), and here taken as part of the binding site. Then, predefined rotamers of the small molecule were rotated around the axis connecting the two coordinating Os, and to a lesser amount around a second axis. Direct contact probabilities between the small molecule and the binding site were calculated. Probabilities for the two coordinating Os were excluded to emphasize contacts for the rest of the molecule. Rotamers and orientations with close intra- or intermolecular contacts were removed using distance criteria, though the plausibility of a rotamer could be evaluated using probability masses for intramolecular contacts.

For each small molecule, the rotamer and orientation with highest probability were found and the probabilities were then used in a logistic regression model that mimics a docking screening task. Two explanatory variables were used in the logistic regression model: the total probability mass of direct contacts, divided by the mass of the molecule (pmPerMass) and the ratio of the number of hydrophobic and hydrophilic fragments (Phob/Phil) in the molecule.

**Figure 18:**
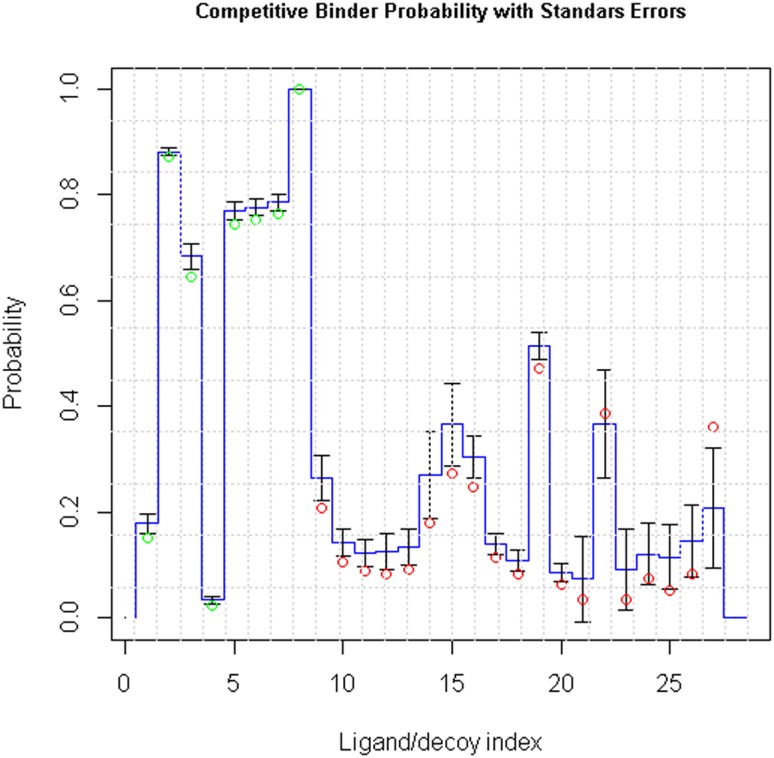
Probabilities calculated for separation of Catechol-O-methyltransferase (COMT) ligands from decoys in a subset of DUD. The green circles represent ligands and the red circles decoys, as they are classified in DUD, when all 27 considered molecules are included in the model. The two extra ligands (see text) have index values 7 and 8 (PDB identifiers LDP and BIA, respectively). Blue step curve gives the mean probability that was obtained from bootstrapping over the two decoy subgroups to calculate standard errors for the logistic regression (error bars representing these are centered at the mean values).

It is well known that in an actual binding affinity calculation for a ligand-protein pair in solution, one needs to consider energetics of direct contacts, water and metal mediated contacts, desolvation and entropy. The variable pmPerMass is here considered to be a measure of the binding energy of direct contacts, whereas the variable Phob/Phil reflects desolvation properties and perhaps tendency for water mediated contacts. Configurational entropy doesn't have in this study any obvious representative, because for the numbers of rotatable bonds (RotBonds) no predictive role was identified. Results of the predictions based on logistic regression are shown graphically in Figure 18. Values for the putative explanatory variables are given in Tables 13, 14 and 15.

The molecule that was most highly ranked, BIA 3-335, is a known tight binding inhibitor [35]. It is also heaviest of the 27 molecules included in the example; approximately 360 hydrogen masses compared to the more typical value that is between 150 and 250. The variable pmPerMass is intensive, i.e., the size of the molecule should not directly influence it's value, and it is assumed that success in predicting relative binding affinities for smaller candidate binders depends strongly on the accuracy of this variable.

**Figure 19:**
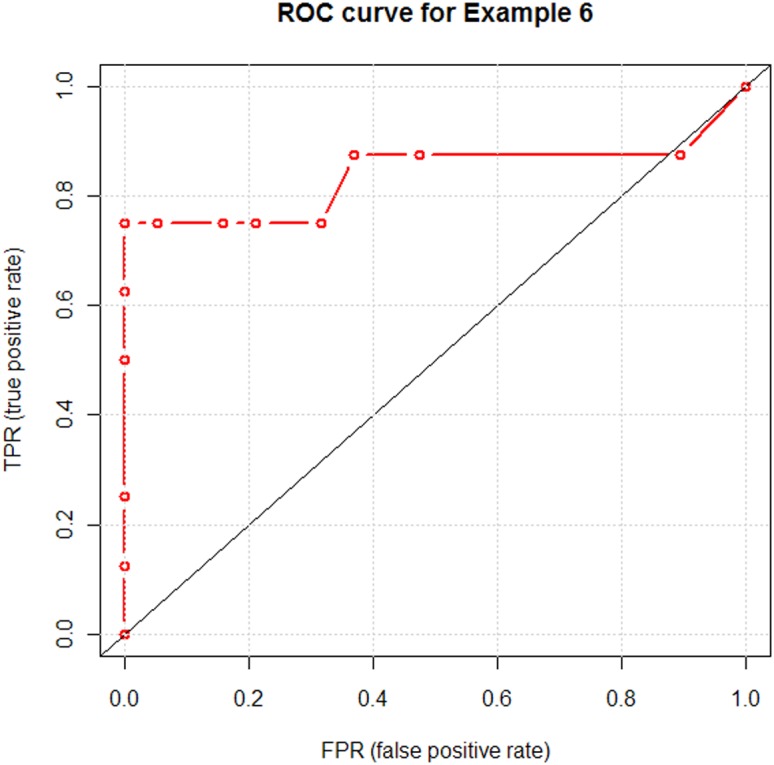
A ROC curve illustrating the functionality of the probabilistic model. The 12 threshold probabilities separating ligands from decoys that were used for calculating the characteristics are 0.05, 0.1, …, 0.3, 0.4, 0.5, 0.65, 0.75, 0.8 and 0.9.

Probabilities derived from the logistic regression are on average over 0.5 for the molecules in the alleged ligand group and below 0.5 for the alleged decoys, which represents a natural threshold between a ligand and a decoy in a screening process. Two exceptions in the ligand group are the low scoring molecules with index values 1 and 4. The ligand with index value 1 has the third highest pmPerMass, but quite low Phob/Phil value, while the ligand with index 4 has a low value for both (see Table 13). Hence, if both these molecules are considered as good binders, our approach does not contain enough information to reveal this. On the other hand, there are not necessarily experimental data available on the binding affinities for the decoys, which in this study were chosen to resemble the ligands as much as possible, each starting with the two, to Mg^2+^ anchoring primary oxygens bonded to an aromatic ring. This means that some decoys might be reasonably good binders. Nevertheless, based on the logistic regression model, molecules in the ligand group have on average clearly higher probabilities (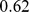) to be ligands than the molecules in the decoy group (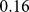). In summary, in the set of 27 chemically similar small molecules, containing 8 experimentally defined ligands, 6 highest ranked molecules were from the ligand group. Consequently, the receiver operating characteristics (ROC) [13] in the screening experiment are: true positive rate TPR = 0.75, false positive rate FPR = 0 and accuracy ACC = 0.93. A ROC curve was produced by using discriminating thresholds having either different TPR or FPR. The ROC curve is given in Figure 19.

One important aspect that has not been considered here, is whether the active form of COMT is a monomer or a multimer. If it is a multimer, it would be interesting to investigate how informative this characteristic is about the properties of suitable ligands. Additional potential molecular characteristics for further study are the flexibility of the binding site and the features of the binding modes having the highest probabilities (pmPerMass).

## Discussion

Predictions about unresolved binding sites, or ligands, can be made by building the preferred contact patterns from the molecules included in a set of functionally classified fragments. In out method, these contact patterns are composed of probability masses calculated for the fragments to have a specific kind of contact in a spatial area. When, for example, the binding affinity of a molecule is studied and the probability masses are defined for an entire molecule, they can be used in a docking and scoring procedure. The absolute binding affinity would be given by the total energetics of the binding process in a thermodynamic setting, including direct and bridged contacts, desolvation and entropy. It is presumed, that using a fragmentation where the fragments have distinct and unique contact patterns, the probability densities described here contain information beyond the chemical complementarity, namely on energetics (for results in this direction, see [36]). This is reasonable by an analogy with quantum mechanics, because it can be argued that the probability masses are proportional to the amount of binding energy, which are needed in evaluating the binding affinities. In our setting this means relative binding affinities, i.e. rankings over a set of ligands and decoys.

The results obtained in Example 6, show a level of reliability that is typical for a successful scoring function, see e.g. reviews [9], [10]. Our experiment revealed that potentially very reliable information could be retrieved when our probabilistic method is combined with an effective search routine. An important aspect is that the ligand and decoy molecules were similar, i.e. the decoys used were ‘drug-like’ [29]. This is based on that they typically had masses between 150 to 250 hydrogen masses, contained both hydrophobic and hydrophilic fragments throughout the structure and were chosen so that each can be anchored to the magnesium ion in the COMT binding site. This should make separation of ligands from decoys challenging and be ultimately based on finer details of the binding affinity, because no decoy was readily rejectable. In a docking and scoring routine such a method can also be used to find the most favourable orientation for the most favourable rotamer, or conformer, of a small molecule in a binding site, i.e. the pose. When adjusting the method for calculations of the absolute binding affinities, the same difficulties will be faced as for any knowledge-based scoring function, see [9].

In addition to the quality of the fragmentation, the reliability of the data is a central issue in the prediction of contact preferences and some issues related to this were discussed in Sections 1 and 2. When choosing the structure for a prediction model, it is essential to understand the data generation process; in principle from the experimental measurement to the coordinate file. Regarding the special characteristics of the experimental method, x-ray diffraction is sensitive to thermal motion in the crystal. This weakens locally the electron density map, and since electron density maps are precisely the starting point in structure refinement, such an effect should preferably be assessed. In the further refinement, constraints are used in order to keep the protein structure within chemically acceptable boundaries. It thus follows that the ligand atom positions have uncertainty which is not straightforward to quantify. Possible approaches to quantification could be exploration of the effects of the constraints on a theoretical basis or using structures refined with different constraints. On the other hand, PDB files contain substantial amounts of metadata that could potentially also be used in modeling. An example of this are the b-factors, which can be used for incorporating thermal motion in the model. Another example is provided by the occupancies that are needed to take into account the more long lasting local displacements, i.e. alternative conformations in the crystallized protein-ligand complexes [8].

Though the results in the example sections are given with standard errors [29], performance of our model in predicting favourable intermolecular contacts could be more quantitatively verified in the future when more extensive reference sets of sufficiently high quality become available. The approximately 10,000 structure files from PDB used as reference data did only give a preliminary test for certain fragment types. This is mainly because of the 3D nature of the problem, since in order to obtain a good spatial resolution, the frequencies need to be defined in less extensive volumes.

Following a reviewer's suggestion, we added a ROC curve to Example 6, which makes it more informative.

## Conclusions

The hierarchies illustrated in our examples show both spatial dependence and reliability. They can also be evaluated quite rapidly from coordinates within a classification, typically in seconds, or in tens of seconds. Thus, tentatively our approach can be used to study structural aspects of biochemical reactions or as a tool in predicting the most favourable binding modes and separating ligands from decoys, as described in the examples. A plausible future test would be to create a hierarchy among a group of ligands and compare their binding probabilities to experimentally measured binding affinities, e.g. those of KiBank database referred to in DUD. Test on each stage of the docking and scoring procedure has to be successfully conducted, before it is shown that the method is applicable for the purpose. Then it can be directly compared, e.g., with the knowledge-based potentials that are only distance dependent.

Reliable evaluation of binding affinities for potential ligands of a binding site, would be a desirable feature of a virtual drug design screen, see for example [9], [37]. As discussed, the distance and direction dependent probability masses obtained with the approach described here, are taken to provide direct information on relative binding affinity, which is supported by the results in Example 6. Regarding further development of our modeling approach, both statistics- and chemistry-based generalizations and improvements are possible, including the obvious expansion to all imaginable molecular fragment types.

Bayesian predictive modeling in the normative sense as defined in [38] provides a potential approach to representing contact preference distributions. Such a predictive model could exploit directly the 3D structures of the probability densities (eq. 3) that model the contact atom positions, instead of considering density parameters as the main characterization of the spatial information. The obvious disadvantage of such an approach is the considerably increased computational effort needed to derive approximations to the sought after predictive distributions.

The reliability of an inferred hierarchy depends, on one hand, on how successfully the error from experimental methods and structure refinement is quantified in terms of the used probability densities. On the other hand it depends on how realistically the chemical likelihood of a contact atom type and the bias in the data set are taken into account. The latter are here incorporated as prior information, see equation (10), which guides the model with chemistry-based knowledge. A third fundamental area for chemistry-based improvements are the classifications (Tables 2 and 3). For example, a classification can be envisioned where the covalent bond count of Atom1 (see Data collection and processing) would be used as one of the characteristics defining the fragment, which would then remove the degeneracy described in section Example 5. This kind of more structural way of defining the fragments would expand the classifications, but should also give fragment definitions that are closer to being unique.

## References

[1] Rantanen V-V, Denessiouk KA, Gyllenberg M, Koski T, Johnson MS (2001) A Fragment Library Based on Gaussian Mixtures Predicting Favorable Molecular Interactions. J Mol Biol 313: 197–214. 11601856 10.1006/jmbi.2001.5023

[2] Rantanen V-V, Gyllenberg M, Koski T, Johnson MS (2003) A Bayesian Molecular Interaction Library. J Comput Aided Mol Des 17: 435–461. 14677639 10.1023/a:1027371810547

[3] Rantanen V-V, Gyllenberg M, Koski T, Johnson MS (2005) A Priori Contact Preferences in Mole- cular Recognition. J Comput Biol Bioinform Res 3: 861–890. 10.1142/s0219720005001417 16078365

[4] Lehtonen JV, Still D-J, Rantanen V-V, Ekholm J, Björklund D, et al (2004) BODIL: A Molecular Modeling Environment for Structure-Function Analysis and Drug Design. J Comput Aided Mol Des 18: 401–419. 15663001 10.1007/s10822-004-3752-4

[5] Kitchen DB, Decornez H, Furr JR, Bajorath J (2004) Docking and Scoring in Virtual Screening for Drug Discovery: Methods and Applications. Nat Rev Drug Discov 3: 935–949. 15520816 10.1038/nrd1549

[6] Scapin G (2006) Structural Biology and Drug Discovery. Curr Pharm Des 12: 2087–2097. 16796557 10.2174/138161206777585201

[7] Berman HM, Westbrook J, Feng Z, Gilliland G, Bhat TN, et al (2000) The Protein Data Bank. Nucleic Acids Res 28: 235–242. 10592235 10.1093/nar/28.1.235 PMC102472

[8] Ravi Acharya K, Lloyd MD (2005) The Advantages and Limitations of Protein Chrystal Structures. Trends Pharmacol Sci 26: 10–14. 15629199 10.1016/j.tips.2004.10.011

[9] Huang S-Y, Grinter SZ, Zou X (2010) Scoring Functions and Their Evaluation Methods for Protein–Ligand Docking: Recent Advances and Future Directions. Phys Chem Chem Phys 12: 12899–12908. 20730182 10.1039/c0cp00151a PMC11103779

[10] Huang S-Y, Zou X (2010) Advances and Challenges in Protein-Ligand Docking. Int J Mol Sci 11: 3016–3034. 21152288 10.3390/ijms11083016 PMC2996748

[11] Mandl F (1988) Statistical Physics, 2nd Ed. Chichester, England: John Wiley & Sons. Chapter 2.

[12] Kortemme T, Morozov AV, Baker D (2003) An Orientation-Dependent Hydrogen Bonding Poten-tial Improves Prediction of Speci.city and Structure for Proteins and Protein–Protein Complexes. J Mol Biol 326: 1239–1259. 12589766 10.1016/s0022-2836(03)00021-4

[13] Egan JP (1975) Signal Detection Theory and ROC Analysis. New York: Academic Press.

[14] Finkelstein AV, Ptitsyn OB (2002) Protein Physics: A Course of Lectures. London, UK: Academic Press.

[15] Desiraju G (1996) The C-H···O Hydrogen Bond: Structural Implications and Supramolecular De- sign. Acc Chem Res 29: 441–449. 23618410 10.1021/ar950135n

[16] Scheinert S, Kar T, Gu Y (2001) Strength of the *C* ^α^ *H*···*O* Hydrogen Bond of Amino Acid Residues. J Biol Chem 276: 9832–837. 11152477 10.1074/jbc.M010770200

[17] Grimme S (2008) Do Special Non-covalent π– π Stacking Interactions Really Exist? Angew Chem Int Ed Engl 47: 3430–3434. 18350534 10.1002/anie.200705157

[18] Auffinger P (2001) Halogen Bonds in Biological Molecules. Proc Natl Acad Sci U S A 101: 16789–6794. 10.1073/pnas.0407607101 PMC529416 15557000

[19] Howard JAK, Hoy VJ, O'Hagan D, Smith GT (1996) How Good is Fluorine as a Hydrogen Bond Acceptor? Tetrahedron 52: 12613–12622.

[20] Dimitropoulos D, Ionides J, Henrick K (2006) Using MSDchem to Search the PDB Ligand Dictionary. Current Protocols in Bioinformatics UNIT 14.3.Available: http://onlinelibrary.wiley.com/doi/10.1002/0471250953.bi1403s15/full. Accessed 9 August 2012. 10.1002/0471250953.bi1403s15 18428761

[21] Gelman A, Carlin JB, Stern HS, Rubin DB (1995) Bayesian Data Analysis. New York: Chapman and Hall.

[22] Bernardo JM, Smith AFM (1994) Bayesian Theory. Chichester, England: Wiley.

[23] Mardia KV, Jupp PE (1999) Directional Statistics, 2nd ed. New York: Wiley.

[24] Mardia KV, Kent JT, Bibby JM (1979) Multivariate Analysis. London: Academic Press.

[25] Guttorp P, Lockhart RA (1988) Finding the Location of a Signal: A Bayesian Analysis. J Am Stat Assoc 83: 322–329.

[26] Nuñez-antonio A, Gutiérrez-peña E (2005) A Bayesian Analysis of Directional Data Using the von Mises-Fisher Distribution. Communications in Statatistics — Simulation and Computation 34: 989–99.

[27] Parr RG, Pearson RG (1983) Absolute Hardness: Companion Parameter to Absolute Electroneg-ativity. J Am Chem Soc 105: 7512–7516.

[28] Gautschi W (1972) Error Function and Fresnel Integrals. In: Abramowitz M, Stegun IA, editors. (1972) Handbook of Mathematical Functions with Formulas, Graphs, and Mathematical Tables. Washington, D.C.: National Bureau of Standards Applied Mathematics Series 55 Tenth Printing. pp. 295–330.

[29] Nicholls A (2008) What Do We Know and When Do We Know It? J Comput Aided Mol Des 22: 239–255. 18253702 10.1007/s10822-008-9170-2 PMC2270923

[30] Strömbergsson H, Kleywegt GJ (2009) A Chemogenomics View on Protein Ligand Spaces. BMC Bioinf 10 ((Suppl 6)) S13. 10.1186/1471-2105-10-S6-S13 PMC2697636 19534738

[31] Drinkwater N, Gee CL, Puri M, Criscione KR, McLeish MJ, et al (2009) Molecular Recognition of Physiological Substrate Noradrenaline by the Adrenaline-Synthesizing Enzyme PNMT and Factors In.uencing Its Methyltransferase Activity. Biochem J 422: 463–471. 19570037 10.1042/BJ20090702 PMC5940352

[32] Bewick V, Cheek L, Ball J (2005) Logistic regression.Crit. Care 9: Statistics review 14. Available: http://ccforum.com/content/9/1/112. Accessed 4 June 2012. 10.1186/cc3045 PMC1065119 15693993

[33] Huang N, Shoichet BK, Irwin JJ (2006) Benchmarking Sets for Molecular Docking. J Med Chem 49: 6789–6801. 17154509 10.1021/jm0608356 PMC3383317

[34] Irwin JJ, Shoichet BK (2005) ZINC–a Free Database of Commercially Available Compounds for Virtual Screening. J Chem Inf Model 45: 177–182. 15667143 10.1021/ci049714 PMC1360656

[35] Bonifácio MJ, Archer M, Rodriques ML, Matias PM, Learmonth DA, et al (2002) Kinetics and Crystal Structure of Catechol-O-Methyltransferase Complex with Co-Substrate and a Novel In- hibitor with Potential Therapeutic Application. Mol Pharmacol 62: 795–805. 12237326 10.1124/mol.62.4.795

[36] Morozov AV, Kortemme T, Baker D (2004) Close Agreement Between the Orientation Dependence of Hydrogen Bonds Observed in Protein Structures and Quantum Mechanical Calculations. Proc Natl Acad Sci U S A 101: 6946–6951. 15118103 10.1073/pnas.0307578101 PMC406446

[37] Gilson MK, Zhou H-X (2007) Calculation of Protein-Ligand Binding Affinities. Annu Rev Biophys Biomol Struct 36: 21–42. 17201676 10.1146/annurev.biophys.36.040306.132550

[38] Geisser S (1993) Predictive Inference: An Introduction. London: Chapman & Hall.

